# Trends in the Hidden Burden of Cancer in an Autopsy-Based Study Over 66 Years in Japan

**DOI:** 10.1001/jamanetworkopen.2025.57812

**Published:** 2026-02-05

**Authors:** Hiroshi Uozaki, Yoshinao Kikuchi, Masato Watanabe, Maiko Tsuchiya, Mariko Yasui, Shiori Watabe, Masahiro Kato

**Affiliations:** 1 Department of Pathology, Teikyo University School of Medicine, Tokyo, Japan

## Abstract

**Importance:**

Conventional cancer statistics account only for individuals with a clinical diagnosis, overlooking potentially large numbers of undetected malignant neoplasms. Autopsy-based studies offer a unique opportunity to estimate the burden of cancer, including latent tumors.

**Objective:**

To evaluate long-term trends and characteristics of cancers using a nationwide autopsy registry in Japan.

**Design, Setting, and Participants:**

This cohort study of hospital-based autopsies over a 66-year period (1958-2023) obtained data from the *Annual of the Pathological Autopsy Cases in Japan *(*APAC-J*), a nationwide database maintained by the Japanese Society of Pathology. Latent cancers, defined as malignant neoplasms undiagnosed during life but discovered at autopsy, were analyzed based on *International Statistical Classification of Diseases, Tenth Revision* and *International Classification of Diseases for Oncology, Third Edition*. All autopsies recorded in *APAC-J* were included. The data were analyzed between May 7 and August 2, 2025.

**Main Outcomes and Measures:**

The main outcome was the proportion of total, multiple, and latent cancers by year, age group, and sex based on trends in latent cancer detection; metastatic status of latent cancers; and the enrichment ratio in autopsy, a novel metric comparing cancer prevalence in autopsies with national mortality statistics.

**Results:**

From 1958 to 2023, 1 486 557 autopsies were registered in *APAC-J* (mean age based on available age group data, 59.1 years; 62.5% male), with 55.2% including cancer diagnoses. The proportion of multiple primary cancers increased from 1.8% (420 of 22 989) in 1974 to 14.4% (957 of 6661) in 2023. The enrichment ratio in autopsy was elevated in adolescents (aged 15-19 years), young adults (aged 20-24 years), and adults aged 80 years or older. Since 1986, 36 133 latent cancers were found in 34 174 of 811 159 autopsies (4.2%). The detection rate of latent cancers increased from 1.7% (683 of 39 839) in 1986 to 7.4% (493 of 6661) in 2023. Latent prostate cancer at age 75 to 79 years was identified in 4.5% (corresponding to a 2017-2021 prevalence of 656.2 per 100 000 population), 6.9-fold higher than the clinical incidence, whereas latent thyroid cancer at age 50 to 54 years showed substantially larger excesses (0.9% of men and 1.7% of women, representing 94.5-fold and 60.7-fold higher prevalences, respectively). Overall, metastases were present in 7.3% of latent cancers (2649 of 36 133).

**Conclusions and Relevance:**

This cohort study of autopsies across Japan found a substantial reservoir of undiagnosed cancer, including some with metastatic potential. These findings highlight the persistent value of autopsy for assessing cancer burden and underscore the need to refine approaches for early detection while minimizing overdiagnosis.

## Key Points

**Question:**

What is the estimated cancer burden in Japan based on nationwide autopsy data over 6 decades?

**Findings:**

In this cohort study of 1 486 557 autopsies performed between 1958 and 2023, 36 133 latent cancers were found. Detection rates increased, with latent prostate and thyroid cancers 6.9-fold and 60-fold more frequent than clinical cases; metastatic latent cancers made up 7.3% of cases and have been increasing in number.

**Meaning:**

These findings suggest that a small reservoir of undiagnosed, metastasis-competent cancers may increase with increasing longevity and underscore the value of autopsy for cancer epidemiology and early detection.

## Teaser

This cohort study evaluates the long-term trends and characteristics of latent cancers based on nationwide autopsy registry data in Japan.

## Introduction

Despite remarkable advances in cancer diagnostics, a substantial proportion of tumors remain undetected during life. These latent, or clinically silent, cancers represent a hidden burden largely unaccounted for in conventional cancer registries. Autopsy remains one of the few methods used to identify such undiagnosed cancers, offering a unique lens into their prevalence and natural history.

The *Annual of the Pathological Autopsy Cases in Japan* (*APAC-J*),^1^ a database maintained by the Japanese Society of Pathology, contains reports of more than 1.48 million nationwide autopsies collected since 1958. This unparalleled resource allows for the investigation of long-term cancer trends over more than 6 decades. Leveraging this comprehensive dataset, we conducted a nationwide analysis to elucidate long-term trends in autopsy-detected cancers and to examine the prevalence, characteristics, and clinical implications of latent tumors.

The nationwide number of autopsies in Japan has steadily declined from approximately 40 000 in the 1980s to just 6557 in 2022.^2^ This decline in autopsy practice is global,^3,4^ making raw numbers less meaningful for comparison. We analyzed proportional trends in autopsy cancer types and compared them with national cancer incidence and mortality data. As autopsy rates vary by age and sex,^2^ these factors were also accounted for.

## Methods

This nationwide, retrospective cohort study examined cancer data from autopsies recorded in all 66 volumes (1958-2023) of *APAC-J*. Neither ethics committee approval nor informed consent was required per the policies of the Teikyo University Medical Research Ethics Committee, as the study relied solely on publicly available published data. This study followed the Strengthening the Reporting of Observational Studies in Epidemiology (STROBE) reporting guideline.

Autopsy records include patient age, sex, clinical diagnoses, and up to 6 pathologic cancer diagnoses per individual, coded according to the *International Statistical Classification of Diseases, Tenth Revision* (organ) and *International Classification of Diseases for Oncology, Third Edition* (histology).^5^ Both fatal cancers and cancers incidentally identified or previously treated were included. From 1986 onward, the database also records latent cancers, defined as malignant neoplasms that were not clinically diagnosed during life but discovered at autopsy.^6,7^ Cancers with unknown primary origin were excluded from the latent cancer analysis. Detailed analyses used autopsies from 1974 onward, when electronic coding began.^8^ For 1958 to 1973, data were taken from published tables; thus, individual-level data were not available.

Nationwide cancer incidence and cause-specific mortality data were obtained from vital statistics and the National Cancer Registry.^9^ We broadly classified cancers into 21 site-based categories consistent with vital statistics groupings (eTable 1 in Supplement 1).

### Statistical Analysis

The data were analyzed between May 7 and August 2, 2025. To compare the representation of specific cancers in autopsy data with national mortality statistics, we developed the enrichment ratio in autopsy (ERA), defined as the ratio of the proportion of a given cancer diagnosed at autopsy to its proportion in national mortality data for the same period. This approach provides a relative measure that is less sensitive to declining autopsy numbers and helps contextualize selection bias in autopsy-based analyses. The ERA is also influenced by undiagnosed, previously treated, or nonfatal cancers. Mean age of individuals with autopsy-detected cancer (MAC) was compared with mean age at death from cancer nationwide (MDN) to examine shifts in age distribution. We also analyzed trends in latent cancer detection from 1986 to 2023, stratified by age, sex, and number of clinical cancers. No statistical significance tests were performed for this study. The analyses were performed using Microsoft Excel for Microsoft 365 (Microsoft Corp).

## Results

From 1958 to 2023, *APAC-J* registered 1 486 557 individuals who underwent autopsy (mean age based on available age group data, 59.1 years; 37.3% female, 62.5% male, and 0.3% of unknown sex). For the detailed analysis period (1974-2023), 1 204 654 individuals who underwent autopsy were included (mean [SD] age, 60.4 [22.8] years; median [IQR] age, 66 [53-75] years; 36.8% female, 62.9% male, and 0.3% of unknown sex). For the latent cancer analysis period (1986-2023), 811 159 individuals who underwent autopsy were registered (mean [SD] age, 63.9 [21.4] years; median [IQR] age, 69 [57-77] years; 35.8% female, 64.0% male, and 0.2% of unknown sex).

### Cancer Incidence

**Figure 1.**
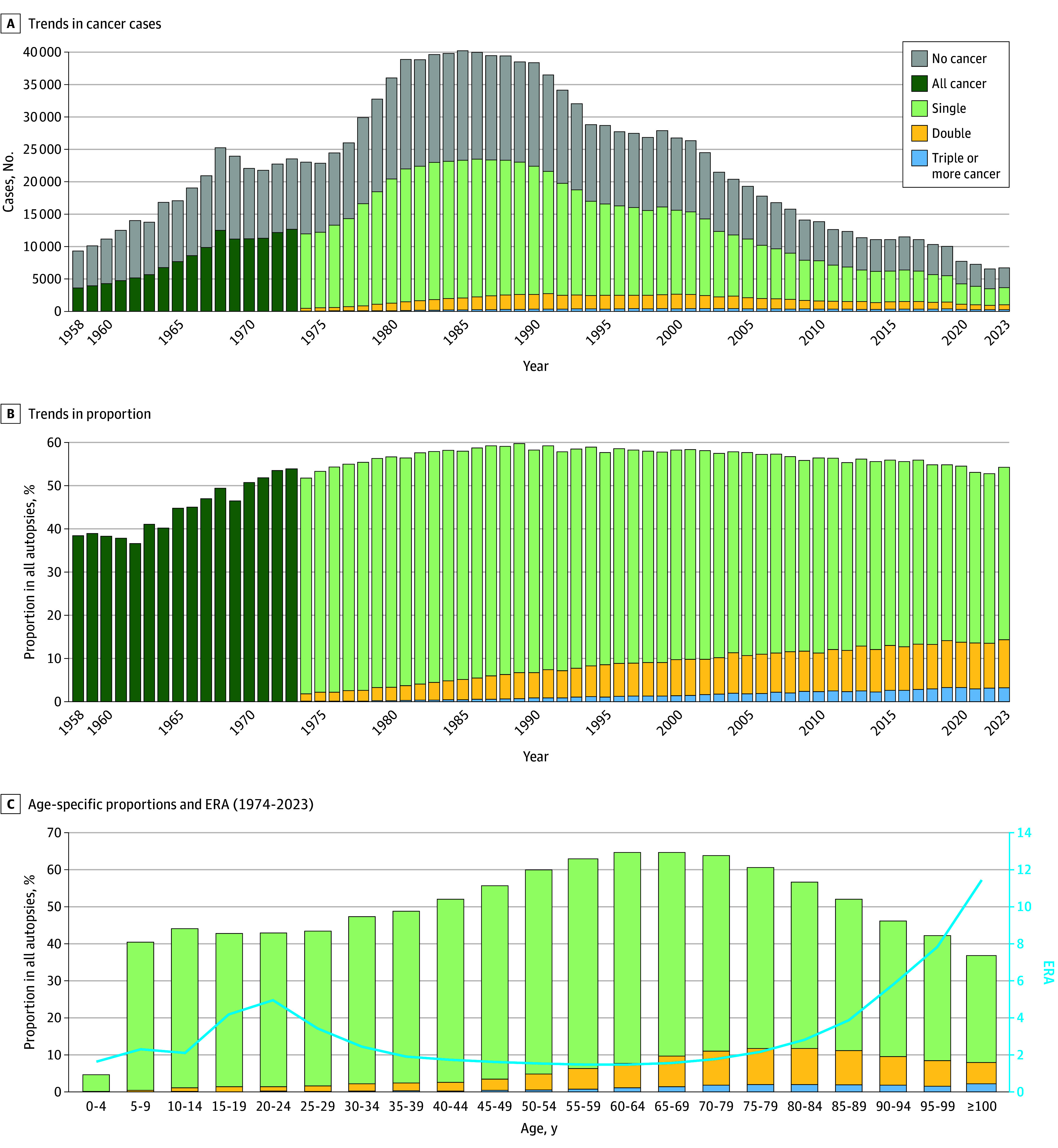
Trends in Autopsy-Detected Cancers Before 1973, multiple primary cancers were not tabulated in published reports. ERA indicates enrichment ratio in autopsy.

From 1958 to 2023, 55.2% of all autopsies included at least 1 cancer diagnosis (Figure 1A). The prevalence of cancer increased from less than 40% in the 1960s to 60% in 1989 and has since slightly declined, remaining greater than 50% (Figure 1B). The proportion of multiple primary cancers increased from 1.8% (420 of 22 989 individuals) in 1974 to 14.4% (957 of 6661 individuals) in 2023.

Figure 1C shows the proportion of autopsy-detected cancer by age group from 1974 to 2023. While the group aged 70 to 74 years had the most autopsy-detected cancers, the proportion of cancers was highest in individuals aged 60 to 69 years (64.6% [70 123 of 108 568]). Individuals aged 100 years or older were rare, accounting for 0.1% (675 of 1 208 005) in 1974 to 2023 and 0.1% (640 of 811 159) in 1986 to 2023 (eFigure 1 in Supplement 1). Age-specific ERA of cancer is also shown in Figure 1C. The ERA exhibited an increase from adolescents aged 15 to 19 years to young adults aged 20 to 24 years, followed by a temporary dip before increasing again in adults aged 80 years or older.

### Cancer Types

**Figure 2.**
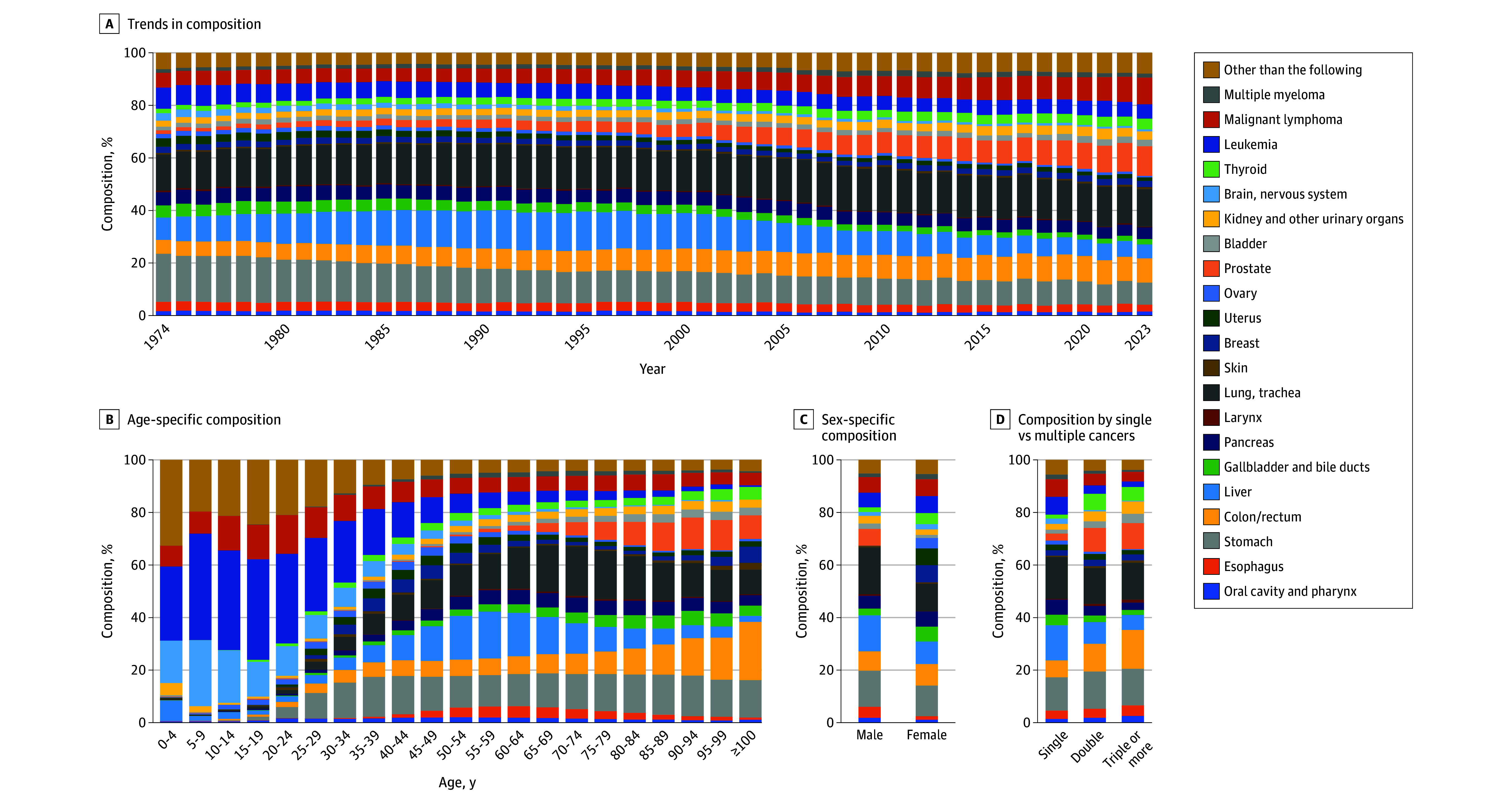
Trends in Autopsy-Detected Cancer Types (1974-2023) Cancers were classified into 21 site-based categories (eTable 1 in Supplement 1). Detailed proportions are provided in eTables 3 to 6 in Supplement 1.

The compositions of the autopsy-detected cancers, summarized by year and age group, are shown in Figure 2A and B and eTables 2 to 4 in Supplement 1. In the trends of cancer type proportions since 1974, stomach cancer was the most common until 1984 (17.9% of all cancers [37 570 of 210 141]). Since 1985, lung cancer was the most prevalent up to 2023 (15.7% of all cancers [91 323 of 582 492]). From 1989 to 1997, the proportion of liver cancer peaked at 14.4% (28 373 of 196 855), but has since decreased. Prostate cancer incidence increased from 1974 to 2023, accompanied by gradual rises in lymphoma and colorectal cancer.

Cancer prevalence varied by age. Leukemia and brain and nervous system tumors were predominant in children and adolescents (aged birth to 19 years). Liver cancer peaked in middle age (up to 17.9% of all cancers [12 858 of 71 906] at age 55-59 years), and lung cancer was most frequent in older adults (up to 18.8% of all cancers [23 959 of 127 238] at age 70-74 years). Gastric cancer maintained a relatively stable prevalence from age 30 years onward (13.3% of all cancers [102 777 of 770 586]).

Figure 2C and eTable 5 in Supplement 1 show the sex-specific cancer composition, with lung and liver cancers more common in male individuals. Figure 2D and eTable 6 in Supplement 1 present cancer types by single vs multiple primaries. Colorectal, prostate, and thyroid cancers were frequent in multiple primaries, while liver cancer, brain tumors, leukemia, and lymphoma were less common.

### Age and Male-to-Female Ratio of Individuals With Autopsy-Detected Cancer

**Figure 3.**
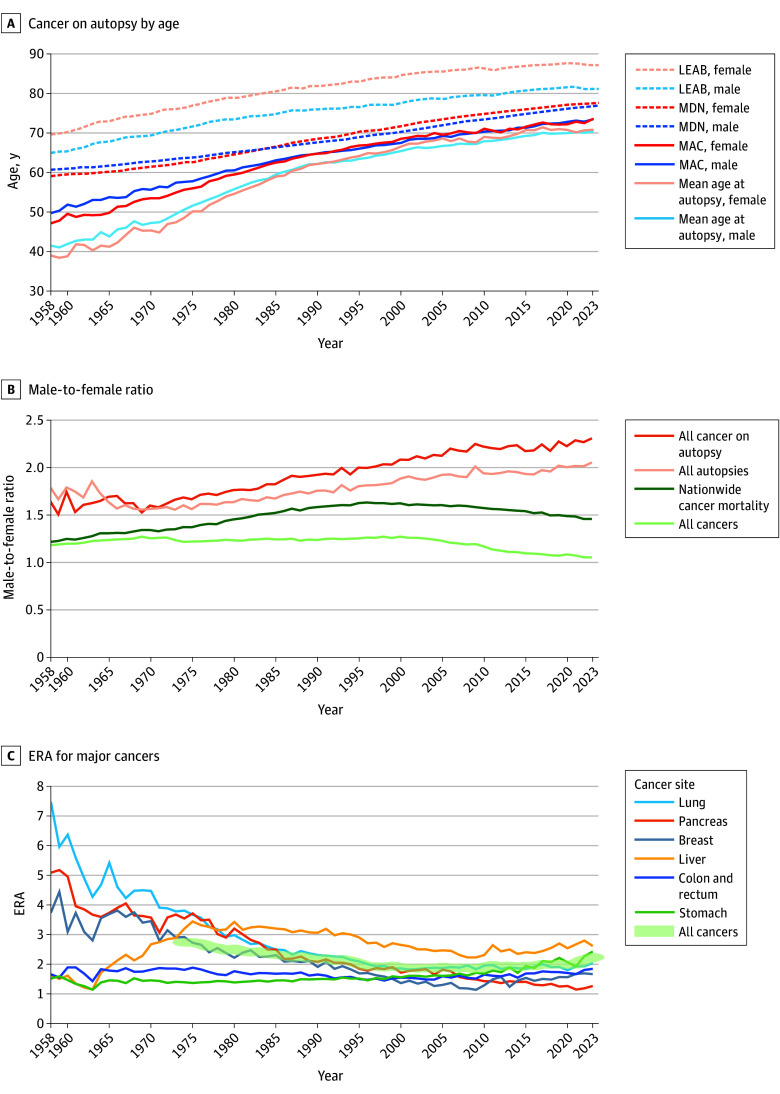
Trends in Age, Male-to-Female Ratio, and Enrichment Ratio in Autopsy (ERA) of Cancer The age and sex ratios of the autopsies were compared with national cancer registry data. LEAB indicates life expectancy at birth; MAC, mean age of individuals with autopsy-detected cancer; MDN, mean age at death from cancer nationwide.

Figure 3A illustrates the trends in MAC, MDN, mean autopsy age, and life expectancy at birth by sex. The MAC data before 1973 were estimated from summary tables. The data from 1965 to 1973 included some benign tumors. The MAC was higher than the mean age of all individuals at autopsy but lower than MDN. All mean ages increased over time, with minimal age differences between sexes. We also examined the trends in MAC and MDN for each of the 21 types of cancer (eFigure 2 in Supplement 1), both of which showed an increasing trend for most cancers.

The trend of the male-to-female ratio for all cancers in the autopsy series is shown in Figure 3B, along with that of cancer deaths from the nationwide vital statistics and in the overall autopsy series. A higher male-to-female ratio was found for cancers in the autopsy series vs the overall series, which increased over time (2.5 in 2023 [male, 2573; female, 1039]). For many types of cancers, the male-to-female ratio gradually increased over time (eFigure 3 in Supplement 1). Meanwhile, the male-to-female ratio for all cancer mortality nationwide declined slightly from 1996 to 2023.

### ERA of Cancer Types

Figure 3C illustrates changes in ERA for the 6 most common life-threatening cancer types in Japan. Pancreatic, lung, and breast cancer had a substantially high ERA (3.9, 4.9, and 3.5, respectively) in the 1960s. Liver cancer showed a notable increase in ERA between 1975 and 1980, followed by a gradual decline thereafter. Gastric cancer and colorectal cancer, although having comparatively lower ERAs, remained stable. The trends in ERA for the 21 types of cancer by decade are shown in eTable 7 in Supplement 1. For brain tumors, which had an extremely high ERA of 34.2 in the 1960s, the ranking has gradually declined since the 1970s (from and ERA of 11.6 to an ERA of 3.7 in the 2020s). Thyroid cancer, leukemia, and lymphoma have continued to remain at the top ranking. Prostate cancer has been gradually rising in rank (from an ERA of 4.1 in the 1960s to an ERA of 8.5 in the 2020s).

### Latent Cancer

**Figure 4.**
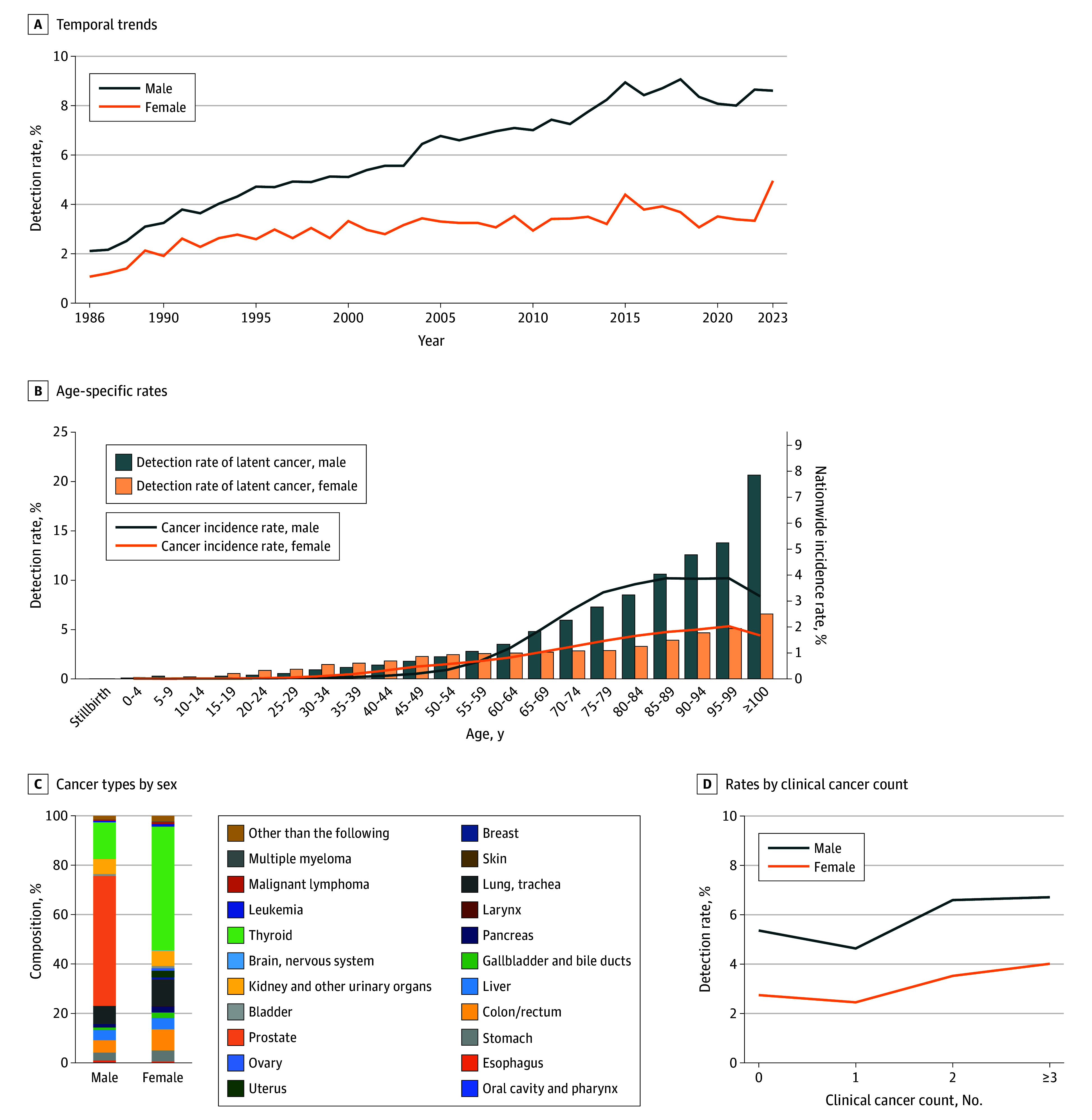
Detection Rates of Latent Cancer B, Data for latent cancers from 1986 to 2023 and the nationwide cancer incidence rates using averaged data from 2017 to 2021. The detailed proportions are provided in eTable 9 in Supplement 1.

Since 1986, 36 133 latent cancers have been registered in 34 174 of 811 159 autopsies (4.2%) (eTable 8 in Supplement 1). The detection rate of latent cancers increased from 1.7% (681 of 39 936 individuals) in 1986 to 7.4% (494 of 6661 individuals) in 2023 (Figure 4A). Latent cancers were more than twice as common in male as in female individuals, with prevalence gradually increasing in both sexes.

Figure 4B shows the detection rate of all latent cancers by age group and sex. The detection rates were similar for both sexes until age 55 to 59 years; thereafter, the rate of latent cancer substantially increased in male individuals (from 2.8% in for those aged 55-59 years to 13.8% for those aged 95-99 years), with more than 20% found in those aged 100 years or older. Figure 4C and eTable 9 in Supplement 1 show the composition of latent cancers by sex.

Figure 4D shows how latent cancer detection varied by the number of clinically diagnosed cancers. In male individuals, the proportion decreased from 5.4% (11 548 of 215 463 individuals) in those with no clinical cancer to 4.6% (12 117 of 261 542 individuals) in those with 1 clinical cancer. The proportion of latent cancers gradually increased in male individuals with 2 or more clinical cancers, reaching 6.7% (420 of 6259) in those with 3 or more clinical cancers. A similar trend was observed in female individuals. The mean age increased slightly by number of clinical cancers (mean [SD] age for male and female individuals, respectively: no clinical cancer, 59.1 [25.7] and 59.5 [28.8] years; 1 clinical cancer, 66.1 [14.0] and 66.6 [15.8] years; 2 clinical cancers, 72.1 [10.5] and 71.4 [12.8] years; ≥3 clinical cancers, 74.6 [9.4] and 74.1 [12.0] years).

**Figure 5.**
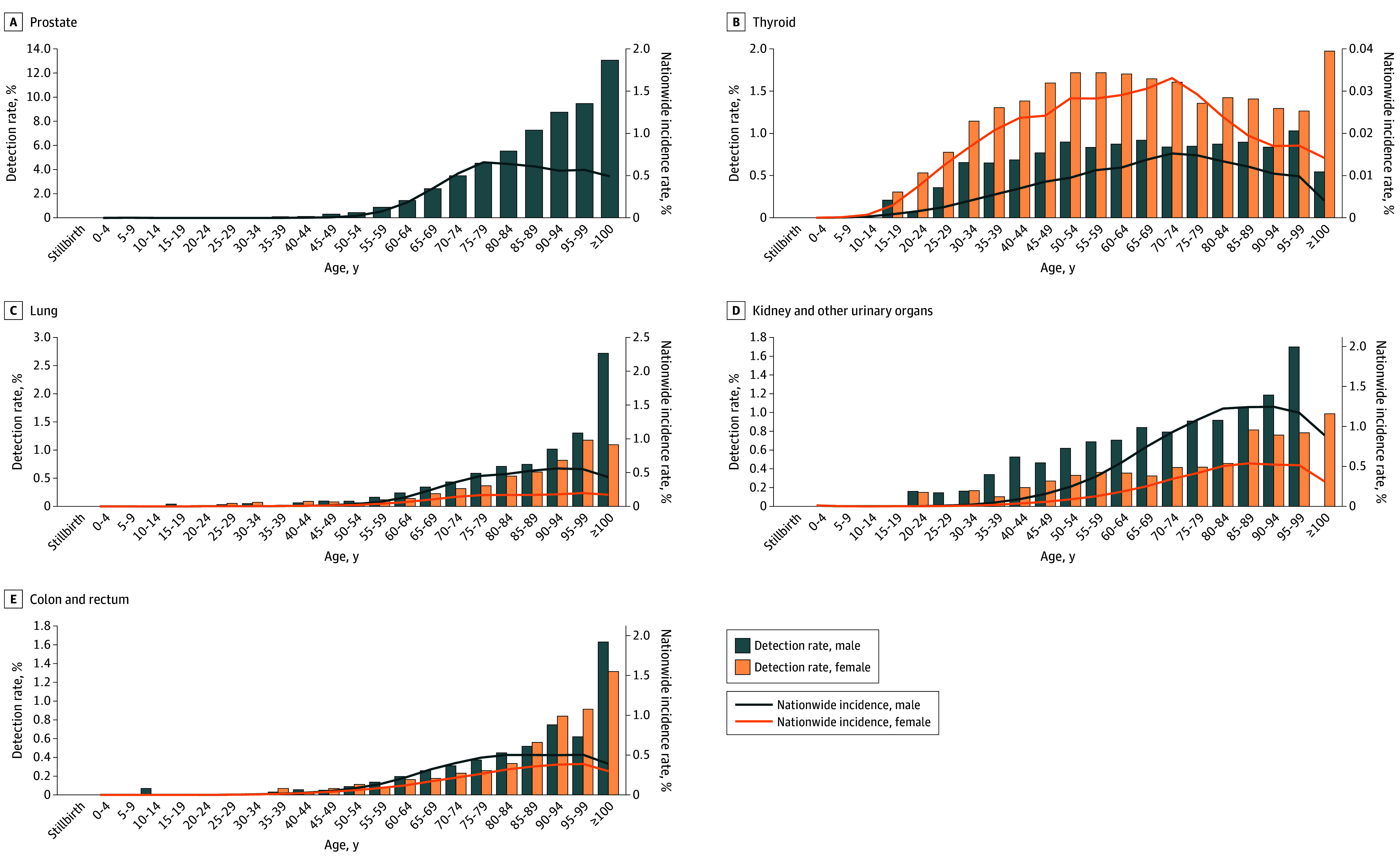
Age-Specific Detection Rates of Latent Cancers With National Cancer Incidence Rates Detection rates of latent cancers are based on 1986-2023 data, whereas incidence rates represent the 2017-2021 mean.

Detection rates for major latent cancers also increased and showed age- and sex-specific patterns (Figure 5; eFigures 4 and 5 in Supplement 1). Latent prostate cancer increased with age, reaching 13.0% (24 of 184 individuals) in men aged 100 years or older. Latent thyroid cancer was more common in female individuals, peaking when aged 50 to 59 years. Latent lung cancer was more frequent in male individuals and exceeded 1% in individuals aged 90 to 99 years. Temporal trends in latent cancer detection rates by sex and age group are shown in eFigure 6 in Supplement 1. Prostate cancer increased across age strata, and thyroid cancer showed a slight upward trend.

The detection rate of latent cancer was compared with the cancer incidence rate in age groups up to 70 to 79 years. At ages 75 to 79 years, latent prostate cancer was identified in 4.5%, corresponding to a 2017-2021 mean prevalence of 656.2 per 100 000 population, or 6.9-fold higher than the clinical incidence. Latent thyroid cancer was detected at age 50 to 54 years in 0.9% of men and 1.7% of women, corresponding to a prevalence of 9.5 and 28.3 per 100 000, or 94.5-fold and 60.7-fold higher than the clinical incidence, respectively. In contrast, among male individuals aged 75 to 79 years, latent lung cancer was identified in 0.6%, kidney cancer in 0.4%, and colorectal cancer in 0.4%, corresponding to prevalences of 539.8, 102.3, and 467.2 per 100 000 population or 1.1-fold, 4.0-fold, and 0.8-fold higher than the clinical incidence, respectively. In female individuals aged 75 to 79 years, latent lung cancer was identified in 0.4%, kidney cancer in 0.2%, and colorectal cancer in 0.3%, corresponding to prevalences of 198.8, 39.4, and 265.2 per 100 000 population or 1.9-fold, 4.7-fold, and 1.0-fold higher than the clinical incidence. For cancers other than thyroid cancer, the prevalence of latent cancer continued to increase to age 100 years or older, whereas the clinical incidence showed only a minimal increase or declined in individuals aged 80 years or older compared with those younger than 80 years.

Overall, 7.3% (2649 of 36 133 individuals) of latent cancers were metastatic, with the lowest rate observed for prostate cancer (2.8% [420 of 14 777 individuals]) and the highest rates for lung (16.7% [478 of 2861 individuals]), pancreatic cancer (27.4% [169 of 617 individuals]), gallbladder and bile duct cancer (21.6% [93 of 430 individuals]), and hematologic cancers (lymphoma, 54.0% [135 of 250 individuals]; multiple myeloma, 31.7% [58 of 183 individuals]; leukemia, 48.6% [35 of 72 individuals]) (eTable 8 in Supplement 1). Overall, 4.0% of latent cancers (n = 1444) had lymph node involvement, and 5.4% (n = 1940) involved distant organs. The metastatic rate for all latent cancers exhibited a slight downward trend over time, decreasing from 8.4% (286 of 3397 individuals) in 1986 to 1989 to 5.5% (114 of 2068) in 2020 to 2023 (eFigure 7 in Supplement 1). This decrease in the metastatic rates persisted even after excluding prostate cancer, for which prevalence among latent cancers has increased.

## Discussion

This cohort study found that the proportion of autopsy-detected cancers in Japan has remained less than 60% since 1958, while detection of multiple cancers has increased. To better interpret trends amid declining autopsy rates and regional differences, we developed ERA, which compares cancer prevalence in autopsies with national mortality data. In age-stratified cancer mortality, the ERA was higher in adolescents and young adults and in adults aged 80 years or older (Figure 1C). Cancers in adolescents and young adults are actively investigated through autopsy. In Japan, senility is often listed as the cause of death in adults aged 80 years or older,^10^ but autopsies frequently revealed other causes, including cancer, which may have contributed to the high ERA in this group.

Both MAC and MDN have increased over the past 66 years, which may reflect the overall rise in life expectancy at birth. The MDN was younger than life expectancy at birth; however, MAC was higher than the autopsy mean age, possibly because autopsies are more often performed in relatively younger individuals.^2^

The rising male-to-female ratio in autopsy-detected cancers mirrored the overall increase seen in all autopsies. As previously reported, male predominance reflects lower autopsy rates in adults older than 75 years, who are predominantly women.^2^ Many major cancers (Figure 3C) had higher incidence and mortality rates in male individuals,^9^ which may have influenced the even higher male-to-female ratio.

The nationwide cancer mortality male-to-female ratio increased until the 1990s but has since declined slightly, possibly due to converging lifestyle habits such as alcohol use and smoking between men and women. Liver cancer findings showed an unusual pattern, which may reflect changes in carcinogenic pathways from hepatitis virus discoveries and improved prevention.

Several cancers exhibited markedly high ERAs in the 1960s, but variation has been relatively stable since the 1990s. Lung and pancreatic cancers had higher ERAs in the 1960s; brain tumors had extremely high values, which may reflect limited imaging accuracy at the time.^11^ The ERAs for gastric and colorectal cancers remained relatively stable, consistent with advances in endoscopy in Japan.^12^ The ERA for liver cancer peaked around 1975, coinciding with intensive research following the discoveries of hepatitis B virus and, subsequently, hepatitis C virus.^13^ For thyroid cancer, leukemia, and prostate cancer, which ranked the highest in ERA, favorable prognosis and latency may contribute.

Most autopsy data worldwide are fragmented, limited to specific regions,^14^ hospitals,^15^ or age groups,^16^ which limits national-level comparisons. Japan’s low immigration, island setting provides unusually comprehensive population-based autopsy data. Autopsy studies from the Netherlands^16^ and Sweden^17^ reported lower proportions of cancers (31% and 39%, respectively) than in this study. In the Dutch report,^16^ autopsy rates for cancer-related or clinically diagnosed cancer deaths were higher than for other causes of death, consistent with our findings that cancers are preferentially examined by autopsy.

Global reports on cancer incidence and mortality are common, but data on latent cancer and true incidence are scarce, often relying on limited cohorts. Data on latent cancer from the *APAC-J* database are unique. This extensive autopsy database includes latent cancers diagnosed through routine pathologic autopsies but lacks explicit examination protocols.^18^ For prostate and thyroid cancer, diagnoses are mostly based on macroscopic examination and a single histologic section. Detection rates vary by method; for example, thin whole-mount sections reveal higher latent prostate cancer rates.^19,20^ Brain examination is limited to just more than 20% of individuals at autopsy.^2^ Bone and soft tissue examination is not systemic. While latent cancers in these sites might be missed, their overall detection may be minimal due to scarce reporting. Trends in noncancer conditions, such as viral hepatitis influencing liver cancer patterns, may be associated with latent cancer prevalence. To account for potential confounding from changes in age structure and sex distribution, latent cancers were analyzed using age- and sex-specific stratification.

Latent cancers were slightly less frequent in patients with a single clinical cancer, suggesting detection or removal during treatment, but their proportion increased in those with multiple primaries, which may reflect genetic, environmental, or age-related predisposition. Latent cancer detection has increased gradually, reaching approximately 9% in male and approximately 4% in female individuals. Increased latent prostate cancer has been reported in other Asian countries.^19,21^ The increase in latent prostate cancer was largely influenced by older age at autopsy, but age-stratified analyses also showed a gradual increase (eFigure 6 in Supplement 1), consistent with a prior report.^22^ Latent thyroid cancer detection increased slightly. Racial differences, as observed for prostate cancer,^19^ may account for discrepancies with a prior meta-analysis.^23^ Autopsy detection methods have changed little, and because autopsy resources remain limited,^24^ most latent cancers are still often diagnosed by hematoxylin-eosin morphology alone. The modest increases may reflect lifestyle and environmental changes.^19^

Although latent cancer was more prevalent in adults aged 80 years or older, clinical incidence decreased with age. While some theories suggest a lower risk of cancers such as lung cancer in older adults,^25^ this study found that only the detection rate for thyroid cancer decreased with age, while the detection rates of other latent cancers increased. The reduced clinical incidence among adults aged 80 years or older may reflect more clinically insignificant cancers or reduced diagnostic efforts due to competing other diseases.

This study reveals a large reservoir of undiagnosed cancer in Japan, including more than 7 times the clinical diagnoses for prostate cancer, 60 times for thyroid cancer, and 4 times for kidney cancer. In this study, latent prostate cancer was detected in 2.8% of autopsies, compared with 20% to 40% reported in step-sectioning studies.^19^ For thyroid cancer, whole-gland sectioning increased detection approximately fourfold.^20^ Collectively, these findings suggest that the estimated reservoir is more than 70-fold greater for prostate cancer and 240-fold greater for thyroid cancer than current incidence rates, and antemortem detection may further exacerbate overdiagnosis.^26,27,28^

In thyroid cancer, the peak incidence of latent cancer in female individuals occurs at age 50 to 59 years, earlier than the clinical peak at age 70 to 79 years. A small fraction of thyroid cancers may progress from latent to clinical over approximately 20 years, a finding consistent with previous reports.^29,30^ More than 98% of thyroid cancers may regress or progress extremely slowly as in a previous report.^31^

Latent lung cancer was detected in 0.4% of autopsies, similar to other studies.^32^ Latent lung and colorectal cancers were only 1.1-fold and 0.8-fold their clinical incidence rates in male individuals, suggesting that most are symptomatic or clinically detected.

Latent cancers are generally unrelated to mortality, but 7.3% showed metastases, indicating meaningful progression in some individuals. Ideally, metastasis should be detected before death. Despite Japan’s high availability of computed tomography screening^33^ and extensive rates of health care use,^34^ some metastatic cancers remain undiagnosed. With increasing prevalence of latent cancer and longer life expectancy, undetected progression represents a growing challenge. Although their current mortality impact is small, missing potentially serious lesions challenges diagnostic accuracy and resource allocation, underscoring the need to improve earlier and efficient detection of latent cancers requiring therapeutic intervention.

### Limitations

This study had some limitations. The *APAC-J*, a unique nationwide database, compiles nearly all autopsies in Japan through voluntary registration supported by reminders and accreditation. Although individual-level details are limited, *APAC-J* offers an overview of autopsy practice. While no explicit autopsy protocol exists, autopsy quality is supported by Japanese Society of Pathology publications^35^ and required autopsy seminars for board certification.^36^

## Conclusions

This cohort study of autopsies performed across Japan found a substantial number of latent cancers, some with metastatic progression. Some metastatic cancers remained undiagnosed during life, even in institutions with established autopsy practices, with a gradual increase over time. These findings underscore the continued value of autopsy in revealing disease burden and informing future cancer detection and control strategies.

## Notes

Supplement 1. **eFigure 1.** Age-Specific Autopsy Numbers (1986-2023) **eFigure 2.** Trends in Mean Age (MAC and MDN) by Cancer Type **eFigure 3.** Trends in MF Ratio for Each Cancer **eFigure 4.** Trends in Detection Rates of Latent Cancers **eFigure 5.** Age-Specific Detection Rates of Latent Cancers **eFigure 6.** Trends in Each Latent Cancer Detection Rate by Age Group **eFigure 7.** Trends in the Rate of Metastasis of Latent Cancers **eTable 1.** Cancer Classification and Corresponding *ICD* codes **eTable 2.** Trends in Cancer Frequencies by Decade **eTable 3.** Trends in Cancer Type Composition, Classified Into 21 Distinct Cancer Types **eTable 4.** Age-Specific Cancer Type Composition **eTable 5.** Cancer Types by Sex **eTable 6.** Cancer Types in Single vs Multiple Primary Cancers **eTable 7.** Changes in ERA Rankings for Cancers by Decade **eTable 8.** Latent Cancer and Metastasis in Autopsies (1986-2023) **eTable 9.** Composition of Latent Cancer by Sex

Supplement 2.
Data Sharing Statement
