# Supplementary material for: Trends in the Hidden Burden of Cancer in an Autopsy-Based Study Over 66 Years in Japan
Source: JAMA Netw Open. 2026 Feb 5;9(2):e2557812. doi: 10.1001/jamanetworkopen.2025.57812 (PMC12878435; doi:10.1001/jamanetworkopen.2025.57812)
Supplement: Supplement 1. — eFigure 1. Age-Specific Autopsy Numbers (1986-2023) eFigure 2. Trends in Mean Age (MAC and MDN) by Cancer Type eFigure 3. Trends in MF Ratio for Each Cancer eFigure 4. Trends in Detection Rates of Latent Cancers eFigure 5. Age-Specific Detection Rates of Latent Cancers eFigure 6. Trends in Each Latent Cancer Detection Rate by Age Group eFigure 7. Trends in the Rate of Metastasis of Latent Cancers eTable 1. Cancer Classification and Corresponding ICD codes eTable 2. Trends in Cancer Frequencies by Decade eTable 3. Trends in Cancer Type Composition, Classified Into 21 Distinct Cancer Types eTable 4. Age-Specific Cancer Type Composition eTable 5. Cancer Types by Sex eTable 6. Cancer Types in Single vs Multiple Primary Cancers eTable 7. Changes in ERA Rankings for Cancers by Decade eTable 8. Latent Cancer and Metastasis in Autopsies (1986-2023) eTable 9. Composition of Latent Cancer by Sex [file jamanetwopen-e2557812-s001.pdf]

Supplemental Online Content

Uozaki H, Kikuchi Y, Watanabe M, et al. Trends in the hidden burden of cancer in an autopsy-based study over 66 years in Japan. *JAMA Netw Open*. 2026;9(2):e2557812. doi:10.1001/jamanetworkopen.2025.57812

eFigure 1. Age-Specific Autopsy Numbers (1986-2023)

eFigure 2. Trends in Mean Age (MAC and MDN) by Cancer Type

eFigure 3. Trends in MF Ratio for Each Cancer

eFigure 4. Trends in Detection Rates of Latent Cancers

eFigure 5. Age-Specific Detection Rates of Latent Cancers

eFigure 6. Trends in Each Latent Cancer Detection Rate by Age Group

eFigure 7. Trends in the Rate of Metastasis of Latent Cancers

eTable 1. Cancer Classification and Corresponding *ICD* codes

eTable 2. Trends in Cancer Frequencies by Decade

eTable 3. Trends in Cancer Type Composition, Classified Into 21 Distinct Cancer Types

eTable 4. Age-Specific Cancer Type Composition

eTable 5. Cancer Types by Sex

eTable 6. Cancer Types in Single vs Multiple Primary Cancers

eTable 7. Changes in ERA Rankings for Cancers by Decade

eTable 8. Latent Cancer and Metastasis in Autopsies (1986-2023)

eTable 9. Composition of Latent Cancer by Sex

This supplemental material has been provided by the authors to give readers additional information about their work.

© 2026 Uozaki H et al. *JAMA Network Open*.

eFigure 1. Age-Specific Autopsy Numbers (1986-2023)

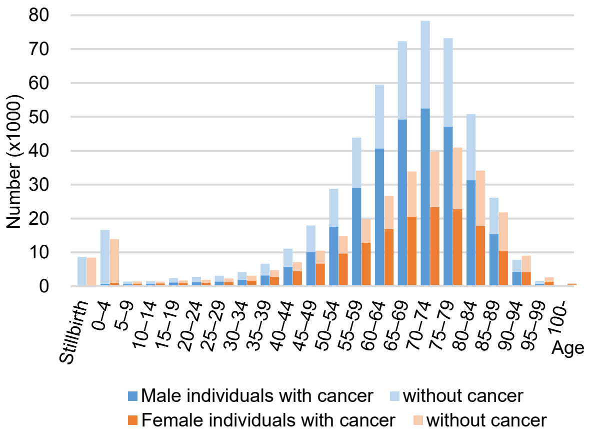

The age distribution of the 811 159 autopsies analyzed for latent cancer from 1986 to 2023 is shown separately for men and women. Individuals aged 100 years or older were rare, comprising 0.08% (640) of the total.

© 2026 Uozaki H et al. *JAMA Network Open*.

eFigure 2. Trends in Mean Age (MAC and MDN) by Cancer Type

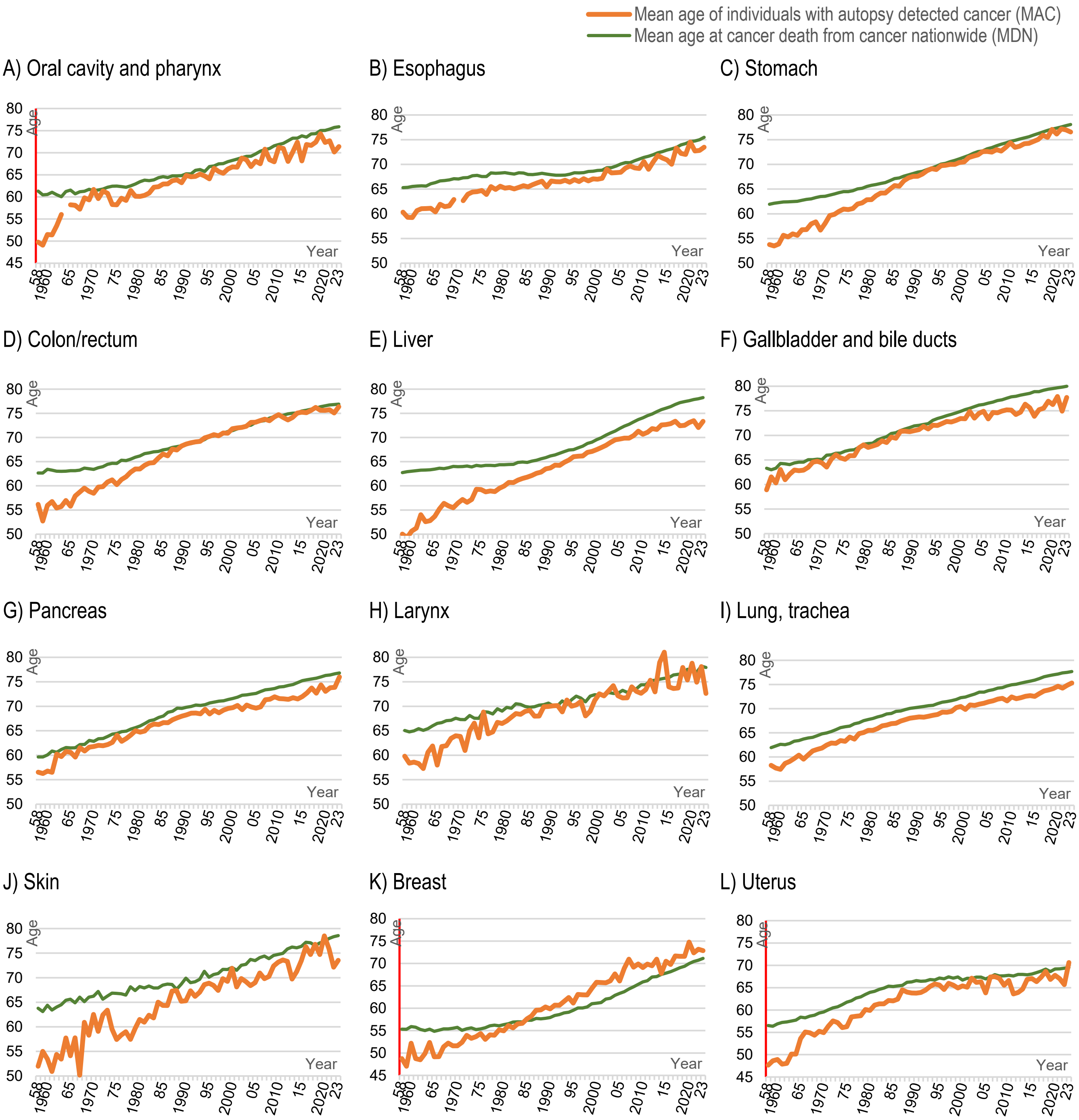

© 2026 Uozaki H et al. *JAMA Network Open*.

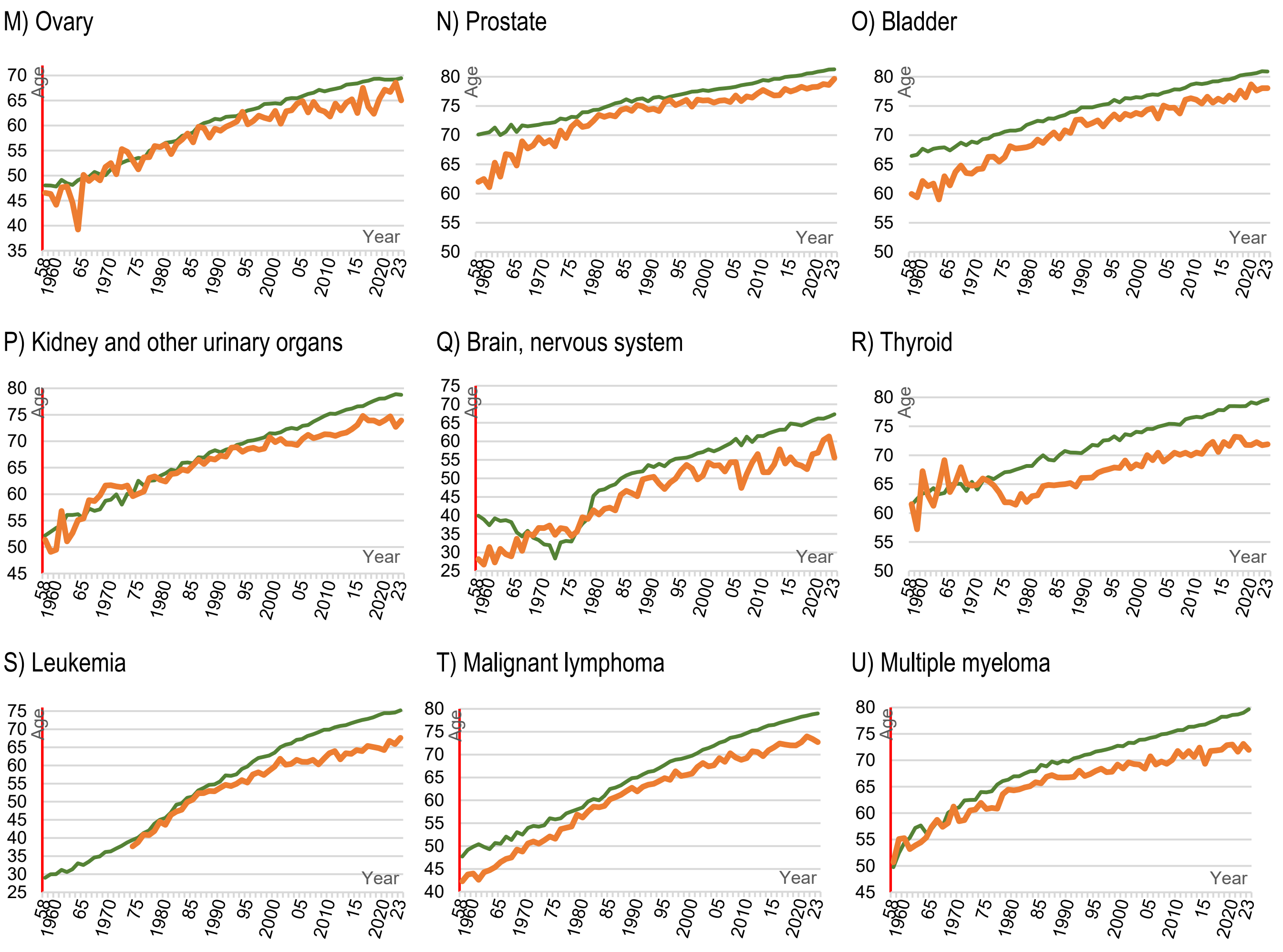

Note that the graph with the red vertical axis has a different scale on the vertical axis compared to the others.

The 1964 data of A and the 1970 data of B were excluded as they were considered outliers. For S, it was difficult to organize data prior to 1973.

© 2026 Uozaki H et al. *JAMA Network Open*.

eFigure 3. Trends in MF Ratio for Each Cancer

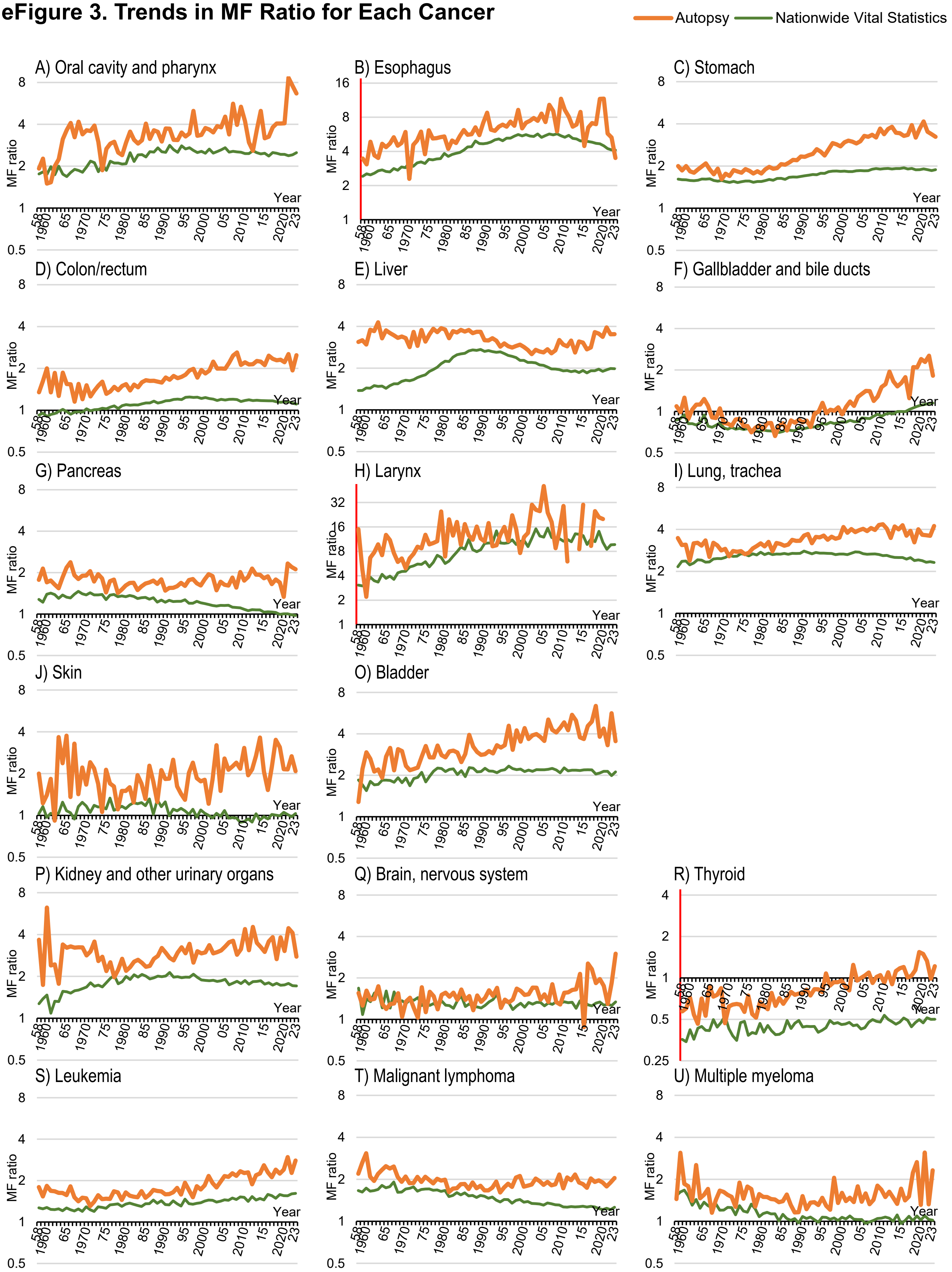

The vertical axis is shown on a logarithmic scale. Note that the graph with the red vertical axis has a different scale on the vertical axis compared to the others.

© 2026 Uozaki H et al. *JAMA Network Open*.

eFigure 4. Trends in Detection Rates of Latent Cancers

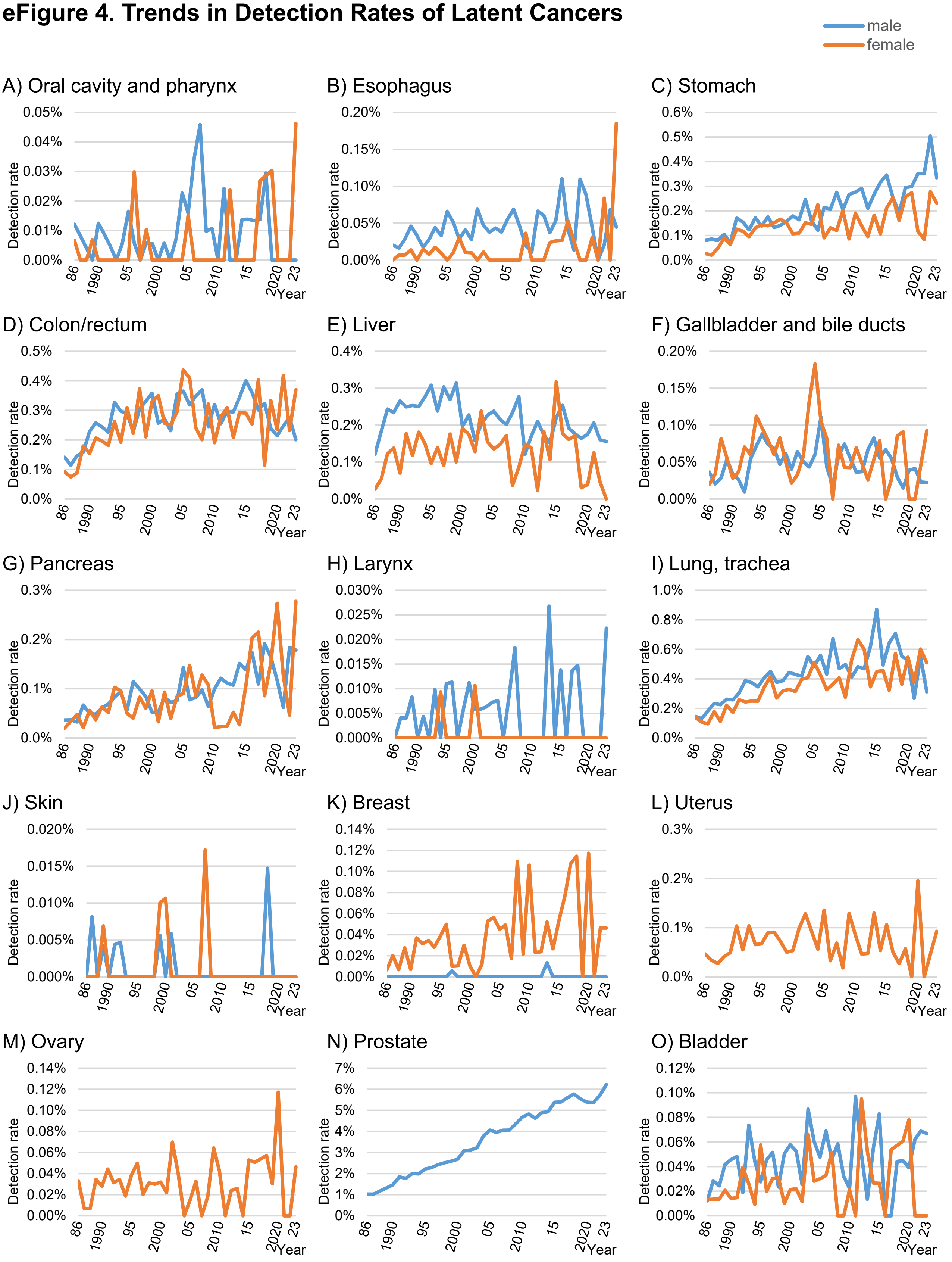

© 2026 Uozaki H et al. *JAMA Network Open*.

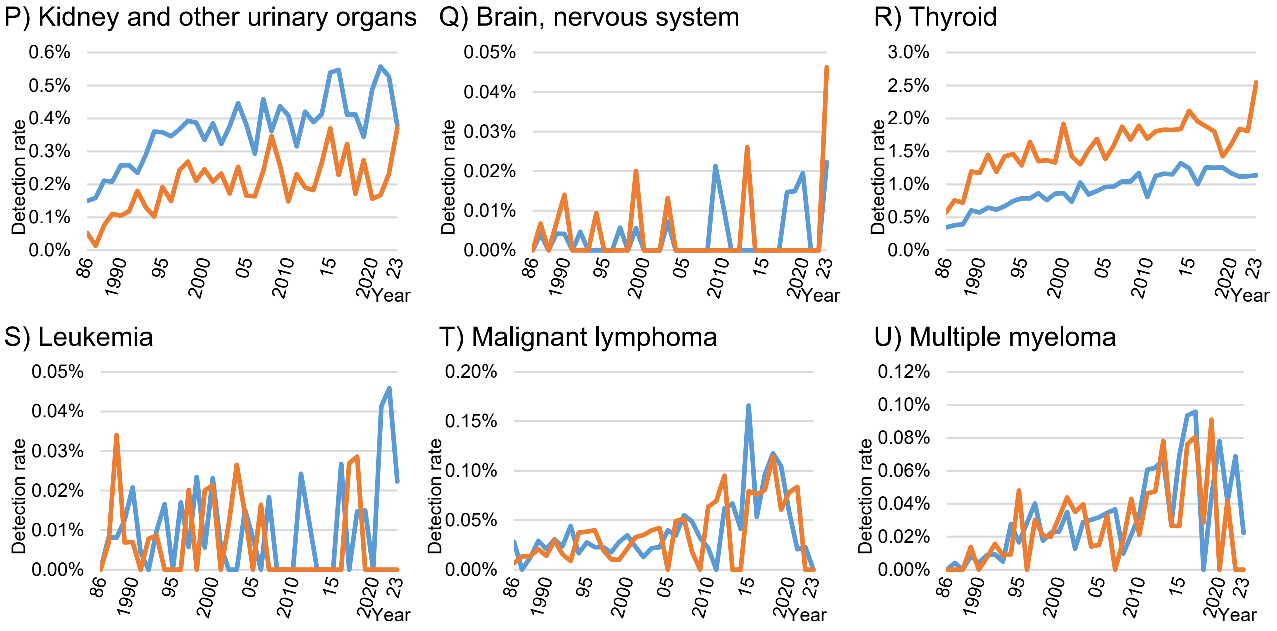

Many major latent cancers are increasing (C,G,I,N,P,R). Colorectal cancer and bile duct cancer are stable (D,F), while liver cancer shows a slight decreasing trend (E).

© 2026 Uozaki H et al. *JAMA Network Open*.

eFigure 5. Age-Specific Detection Rates of Latent Cancers

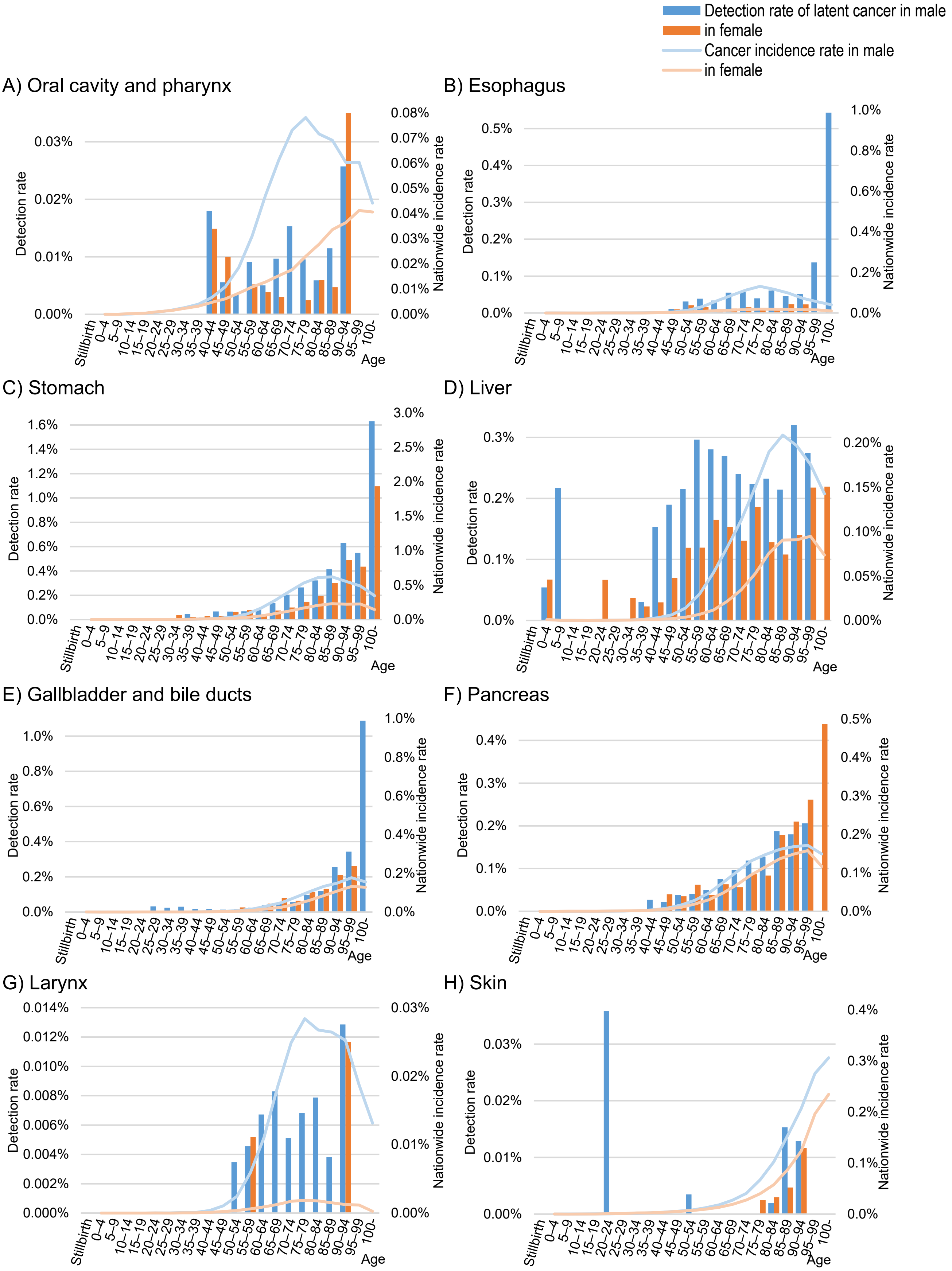

© 2026 Uozaki H et al. *JAMA Network Open*.

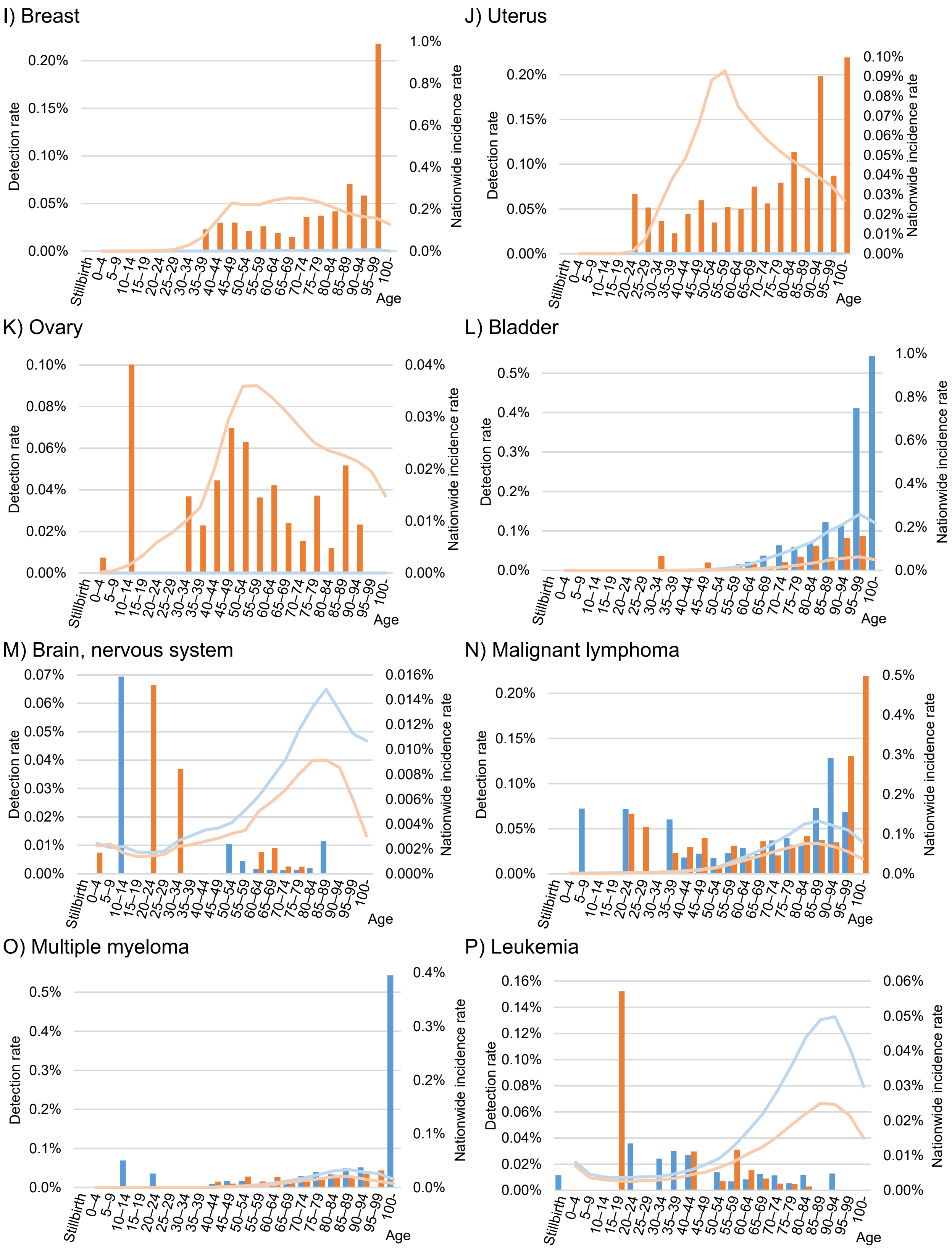

Detection rates are based on 1986–2023 data. The incidence rates represent average data from 2017–2021.

© 2026 Uozaki H et al. *JAMA Network Open*.

eFigure 6. Trends in Each Latent Cancer Detection Rate by Age Group

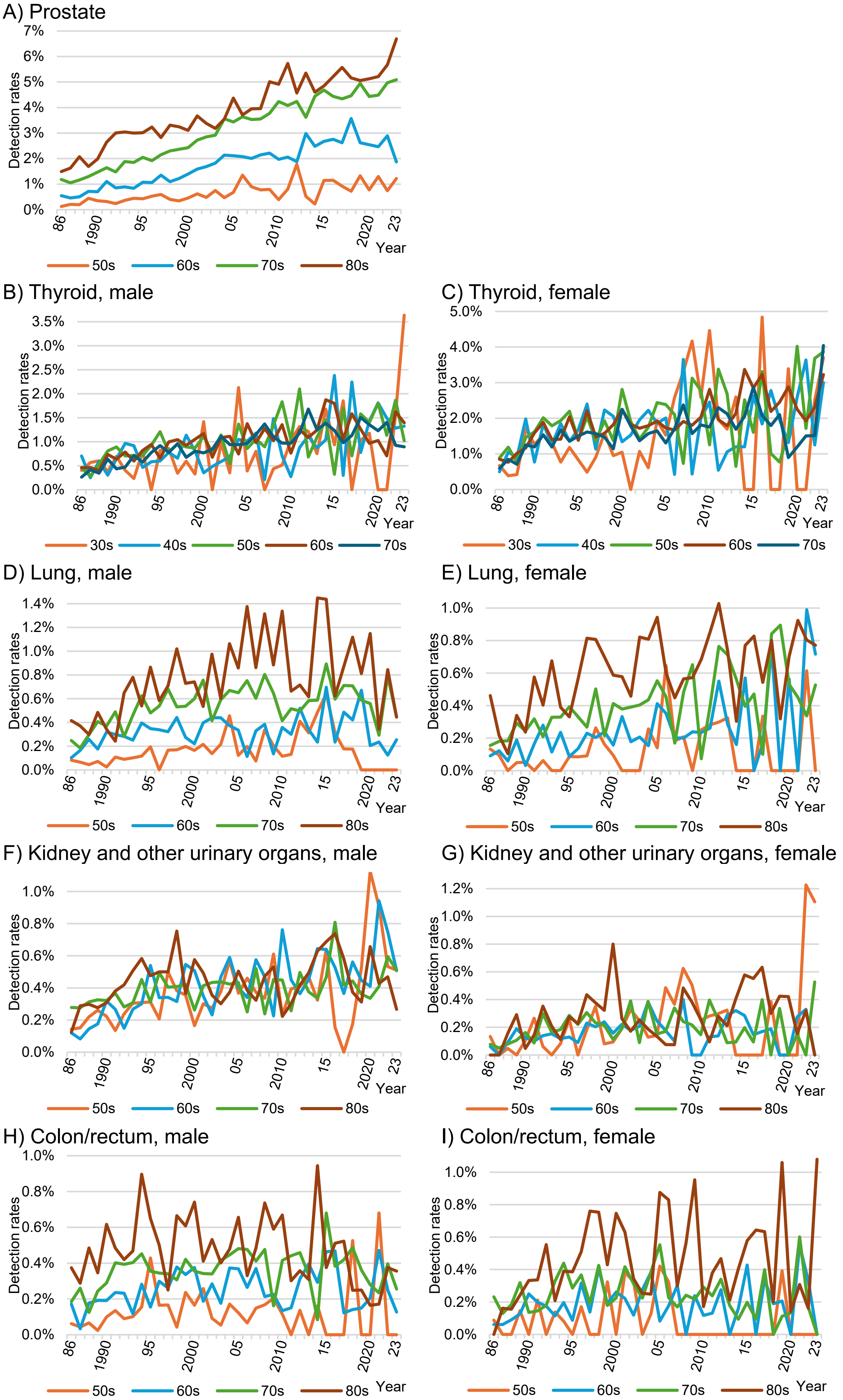

Latent prostate cancer increased across all age groups, whereas latent thyroid cancer showed a slight increase. For lung, renal, and colorectal cancers, numbers were small within age strata, and no clear temporal trends were observed.

© 2026 Uozaki H et al. *JAMA Network Open*.

eFigure 7. Trends in the Rate of Metastasis of Latent Cancers

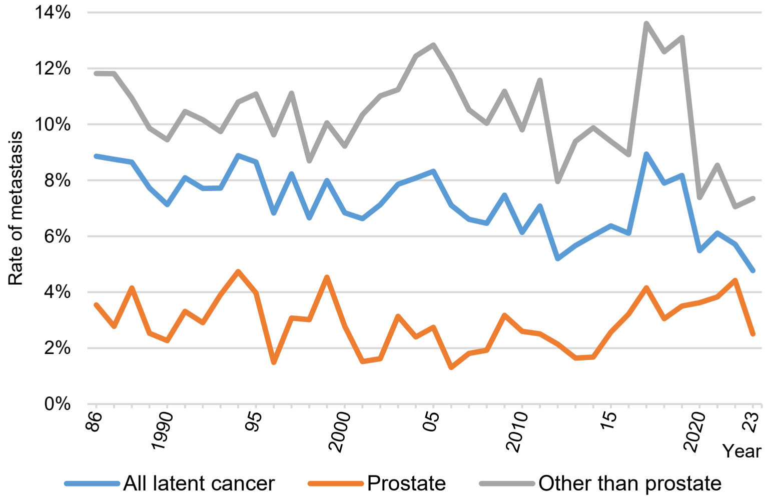

The proportion of metastatic cancers among all latent cancers has slightly declined over time. Metastasis in latent prostate cancer is relatively rare, at approximately 3%, and has remained steady.

© 2026 Uozaki H et al. *JAMA Network Open*.

eTable 1. Cancer Classification and Corresponding *ICD* Codes

Site of cancer	*ICD-9* (1974–1992)	*ICD-10* (1993–2023)	Proportion in cancers of *APAC-J*
Oral cavity and pharynx	140–149	C00–C14	1.6%
Esophagus	150	C15	3.2%
Stomach	151	C16	13.1%
Colon/rectum	153–154	C18–C20	7.6%
Liver	155, 199.1C	C22	12.0%
Gallbladder and bile ducts	156	C23–C24	3.6%
Pancreas	157	C25	5.2%
Larynx	161	C32	0.4%
Lung, trachea	162	C33–C34	15.4%
Skin	172–173	C43–C44	0.5%
Breast	174–175	C50	2.2%
Uterus	179–182	C53–C55	2.1%
Ovary	183	C56	1.3%
Prostate	185	C61	4.3%
Bladder	188	C67	1.8%
Kidney and other urinary organs	189	C64–C66, C68	2.6%
Brain, nervous system	191–192	C70–C72	1.6%
Thyroid	193	C73	2.6%
	**ICD-9**	**ICD-O**	
Leukemia	204.0–209.7	980–996, 998	5.9%
Malignant lymphoma	200.0–202.9	959–972,9767	6.1%
Multiple myeloma	203.8, 203.9	973, 9761–9765	1.6%

© 2026 Uozaki H et al. *JAMA Network Open*.

eTable 2. Trends in Cancer Frequencies by Decade

	1960s	1970s	1980s	1990s	2000s	2010s	2020s[a]
1	Stomach 9.3%[b]	Stomach 10.1%	LungT 9.6%	LungT 10.5%	LungT 10.8%	LungT 10.8%	LungT 9.8%
2	LungT 5.3%	LungT 7.5%	Stomach 9.5%	Liver 9.6%	Liver 7.9%	Stomach 6.9%	Prostate 7.6%
3	Leukemia 3.9%	Leukemia 5.7%	Liver 8.2%	Stomach 8.3%	Stomach 7.8%	ColonR 6.3%	Lymphoma 6.6%
4	Liver 2.3%	Liver 4.9%	ColonR 4.3%	ColonR 5.2%	ColonR 5.8%	Prostate 6.2%	ColonR 6.2%
5	Lymphoma 2.1%	ColonR 2.9%	Leukemia 3.9%	Lymphoma 3.8%	Lymphoma 4.7%	Lymphoma 5.8%	Stomach 6.0%
6	Uterus 2.0%	Lymphoma 2.8%	Pancreas 3.4%	Leukemia 3.7%	Prostate 4.5%	Liver 5.6%	Liver 4.3%
7	BrainNS 1.7%	Pancreas 2.7%	Lymphoma 3.3%	Pancreas 3.4%	Leukemia 3.7%	Leukemia 3.8%	Leukemia 4.0%
8	ColonR 1.7%	GBBD 2.5%	GBBD 2.7%	Prostate 2.7%	Pancreas 3.6%	Pancreas 3.4%	Pancreas 3.2%
9	Pancreas 1.6%	Esophagus 1.8%	Esophagus 2.1%	GBBD 2.3%	Esophagus 2.2%	Thyroid 2.5%	Thyroid 2.7%
10	GBBD 1.6%	Uterus 1.7%	Prostate 1.6%	Esophagus 2.2%	Thyroid 2.1%	KidneyU 2.2%	KidneyU 2.4%
11	Esophagus 1.5%	BrainNS 1.5%	Uterus 1.6%	Thyroid 1.9%	KidneyU 2.0%	Esophagus 1.9%	Esophagus 2.0%
12	Breast 1.0%	Breast 1.3%	KidneyU 1.5%	KidneyU 1.8%	GBBD 2.0%	GBBD 1.6%	Bladder 1.8%
13	OralCP 0.9%	KidneyU 1.1%	Breast 1.4%	Breast 1.5%	Myeloma 1.3%	Breast 1.5%	Breast 1.7%
14	Bladder 0.7%	OralCP 1.0%	Thyroid 1.4%	Uterus 1.3%	Bladder 1.3%	Myeloma 1.5%	GBBD 1.3%
15	KidneyU 0.7%	Thyroid 0.9%	BrainNS 1.4%	BrainNS 1.2%	Breast 1.3%	Bladder 1.4%	Myeloma 1.2%
16	Ovary 0.6%	Bladder 0.9%	OralCP 1.2%	OralCP 1.2%	OralCP 1.1%	Uterus 1.2%	Uterus 1.1%
17	Thyroid 0.4%	Ovary 0.9%	Bladder 1.1%	Bladder 1.1%	Uterus 1.1%	OralCP 1.0%	OralCP 1.0%
18	Myeloma 0.4%	Prostate 0.8%	Ovary 1.0%	Myeloma 1.1%	BrainNS 1.0%	BrainNS 0.9%	BrainNS 0.7%
19	Prostate 0.4%	Myeloma 0.7%	Myeloma 1.0%	Ovary 0.9%	Ovary 0.8%	Ovary 0.7%	Ovary 0.6%
20	Larynx 0.3%	Larynx 0.3%	Skin 0.4%	Skin 0.4%	Skin 0.5%	Skin 0.6%	Skin 0.5%
21	Skin 0.2%	Skin 0.3%	Larynx 0.3%	Larynx 0.3%	Larynx 0.3%	Larynx 0.2%	Larynx 0.2%

a: 2020–2023, b: proportion in all autopsies.

Abbreviations: LungT: Lung, trachea, ColonR: Colon/rectum, BrainNS: Brain, nervous system, KidneyU: Kidney and other urinary organs, GBBD: Gallbladder and bile ducts, OralCP: Oral cavity and pharynx.

© 2026 Uozaki H et al. *JAMA Network Open*.

eTable 3. Trends in Cancer Type Composition, Classified Into 21 Distinct Cancer Types

Year	Other than the following	Multiple myeloma	Malignant lymphoma	Leukemia	Thyroid	Brain, nervous system	Kidney and other urinary organs	Bladder	Prostate	Ovary	Uterus	Breast	Skin	Lung, trachea	Larynx	Pancreas	Gallbladder and bile ducts	Liver	Colon/rectum	Stomach	Esophagus	Oral cavity and pharynx
1974	6.2	1.3	5.7	8.1	1.6	2.9	2.2	1.5	1.3	1.7	3.2	2.3	0.6	13.8	0.6	5.0	4.6	8.5	5.3	18.3	3.6	1.6
1975	5.7	1.2	5.3	7.6	1.7	2.9	2.2	1.6	1.4	1.9	3.1	2.3	0.6	14.0	0.6	5.4	4.9	9.4	5.6	17.3	3.6	1.8
1976	5.5	1.3	5.4	8.0	1.8	2.6	2.3	1.7	1.3	1.7	3.1	2.2	0.6	14.3	0.6	5.2	4.3	9.6	5.6	17.4	3.4	1.8
1977	5.5	1.3	5.4	7.4	1.8	2.4	2.2	1.9	1.4	1.6	2.9	2.2	0.6	14.3	0.5	5.6	4.8	9.9	5.6	17.6	3.3	1.8
1978	5.2	1.3	5.2	7.1	1.8	2.3	2.2	1.8	1.7	1.8	3.0	2.4	0.5	14.7	0.4	5.1	4.9	10.2	5.7	17.5	3.6	1.7
1979	5.1	1.3	5.5	7.1	1.8	2.2	2.4	1.8	1.9	1.7	3.0	2.2	0.5	14.3	0.5	5.1	4.8	10.8	5.7	17.4	3.2	1.6
1980	4.9	1.3	5.4	6.5	1.9	2.3	2.3	1.9	2.0	1.6	2.9	2.1	0.5	14.7	0.4	5.8	4.6	11.5	6.1	16.0	3.4	1.8
1981	4.7	1.4	5.6	6.6	1.8	2.1	2.2	1.7	2.1	1.6	2.6	2.4	0.5	15.0	0.5	5.7	4.6	11.3	6.3	16.1	3.4	1.8
1982	4.4	1.4	5.3	6.2	1.9	2.2	2.4	1.8	2.2	1.8	2.6	2.4	0.5	14.9	0.4	5.6	4.4	12.2	6.4	15.7	3.4	1.8
1983	4.5	1.5	5.2	6.1	2.3	2.2	2.5	1.7	2.2	1.6	2.5	2.1	0.5	15.1	0.4	5.4	4.5	12.5	6.5	15.3	3.4	1.9
1984	4.5	1.5	5.1	6.2	2.2	1.9	2.6	1.7	2.4	1.5	2.7	2.3	0.5	15.3	0.5	5.0	4.4	12.9	6.8	14.9	3.3	1.8
1985	4.3	1.5	4.9	5.9	2.3	2.0	2.4	1.7	2.5	1.6	2.4	2.4	0.5	15.1	0.4	5.4	4.5	13.4	6.8	14.8	3.5	1.6
1986	4.2	1.6	5.2	6.2	2.3	1.9	2.4	1.7	2.8	1.4	2.3	2.2	0.5	15.3	0.5	5.0	4.4	13.6	7.1	14.3	3.5	1.7
1987	4.2	1.5	5.2	6.0	2.5	2.1	2.5	1.5	2.8	1.4	2.4	2.2	0.6	15.1	0.4	5.3	4.1	13.8	7.3	13.8	3.4	1.7
1988	4.4	1.6	5.1	5.7	2.4	1.9	2.5	1.6	2.9	1.4	2.2	2.2	0.5	15.8	0.4	5.4	3.9	13.9	7.5	13.7	3.3	1.7
1989	4.3	1.6	5.2	5.8	2.6	1.8	2.3	1.6	3.1	1.3	2.2	2.3	0.5	16.0	0.5	5.2	3.7	14.1	7.4	13.5	3.0	1.7
1990	4.5	1.5	5.2	5.7	2.6	1.7	2.4	1.6	3.3	1.3	2.3	2.1	0.4	15.9	0.5	5.1	3.6	14.5	7.8	13.0	3.2	1.5
1991	4.4	1.6	5.4	5.3	2.7	1.5	2.6	1.6	3.4	1.3	2.0	2.4	0.5	15.8	0.5	5.3	3.5	14.7	7.6	12.7	3.3	1.8
1992	4.3	1.6	5.6	5.5	2.6	1.7	2.4	1.8	3.6	1.4	1.9	2.1	0.5	16.5	0.4	5.2	3.5	14.4	7.6	12.6	3.1	1.6
1993	4.4	1.4	5.6	6.0	2.6	1.6	2.7	1.6	3.7	1.1	1.8	2.2	0.5	16.1	0.4	5.1	3.7	14.4	7.7	12.3	3.3	1.6
1994	4.6	1.6	5.6	5.7	2.8	1.7	2.5	1.8	3.8	1.4	1.9	2.2	0.5	16.0	0.5	5.0	3.5	14.5	7.9	12.0	3.1	1.5
1995	4.7	1.3	5.7	5.8	2.6	1.6	2.7	1.6	4.2	1.2	2.0	2.2	0.5	15.7	0.4	4.8	3.6	14.9	7.9	12.1	3.2	1.5
1996	4.7	1.6	6.1	5.2	2.8	1.4	2.7	1.6	4.1	1.2	1.8	2.2	0.6	16.0	0.6	4.8	3.3	14.2	8.1	11.8	3.4	1.8
1997	4.9	1.7	6.0	5.0	2.8	1.4	2.7	1.7	4.1	1.2	1.7	2.2	0.5	15.4	0.5	5.1	3.3	14.2	8.4	12.1	3.5	1.7
1998	4.7	1.6	6.1	5.2	2.7	1.4	2.8	1.8	4.6	1.3	1.7	2.1	0.5	15.8	0.4	5.1	3.5	13.6	8.1	11.7	3.4	1.8

© 2026 Uozaki H et al. *JAMA Network Open*.

1999	4.8	1.6	6.3	5.5	3.0	1.4	2.9	1.9	4.7	1.3	1.7	2.0	0.5	14.8	0.4	5.3	3.3	13.6	8.4	11.9	3.2	1.6
2000	5.1	1.6	6.5	5.2	2.9	1.2	2.8	1.8	4.9	1.2	1.7	1.9	0.5	15.3	0.4	4.9	3.2	13.5	8.6	11.6	3.6	1.7
2001	5.3	1.7	6.2	5.0	2.7	1.2	2.7	1.9	5.2	1.1	1.7	2.1	0.6	15.3	0.5	5.1	3.3	13.3	8.7	11.8	3.3	1.6
2002	5.2	1.8	6.9	5.1	2.9	1.1	2.6	1.8	5.7	1.1	1.6	1.9	0.6	15.5	0.4	5.3	2.9	12.7	8.7	11.5	3.3	1.4
2003	5.5	1.7	6.6	5.3	3.0	1.2	2.9	1.9	5.9	1.1	1.7	2.0	0.4	15.5	0.4	5.5	3.0	12.1	8.7	10.9	3.3	1.5
2004	5.5	1.8	6.9	5.0	3.0	1.1	3.1	2.1	6.4	0.8	1.7	1.8	0.6	15.9	0.4	5.0	2.9	11.5	8.5	11.1	3.5	1.6
2005	5.7	1.8	6.9	5.7	2.9	0.9	2.8	1.8	6.7	1.2	1.5	1.9	0.6	15.7	0.4	5.5	2.8	11.1	8.9	10.6	3.1	1.5
2006	6.6	1.7	6.6	5.5	3.0	1.0	2.7	1.9	7.0	1.0	1.3	2.0	0.4	16.3	0.4	5.4	2.7	10.8	8.7	10.8	3.0	1.2
2007	6.5	2.2	7.0	5.7	3.2	1.1	2.8	1.7	6.9	1.2	1.4	1.7	0.6	16.2	0.5	4.9	2.6	9.8	9.1	10.7	2.9	1.3
2008	7.1	2.0	7.6	5.3	3.1	1.1	3.1	2.1	7.3	1.0	1.4	1.7	0.5	16.7	0.4	4.9	2.4	9.3	8.6	10.0	3.0	1.4
2009	6.7	2.0	7.8	5.5	3.4	0.9	3.1	1.8	7.7	1.1	1.5	1.7	0.8	16.0	0.3	5.0	2.5	9.2	8.4	10.5	2.8	1.1
2010	6.8	2.0	7.5	5.4	2.8	0.9	3.1	1.6	7.9	1.1	1.6	1.9	0.7	16.9	0.3	4.8	2.4	9.0	9.1	10.0	2.9	1.2
2011	6.6	2.2	8.2	5.4	3.6	0.8	2.7	1.9	8.1	1.1	1.5	2.2	0.6	16.0	0.3	4.6	2.2	9.4	8.7	9.8	2.8	1.3
2012	7.1	2.1	8.0	5.1	3.5	1.0	2.9	1.8	8.3	1.1	1.7	2.3	0.8	15.6	0.4	4.6	2.5	8.6	9.0	9.6	2.8	1.0
2013	7.1	1.9	8.1	5.4	3.2	0.8	3.1	2.1	8.6	1.0	1.6	1.8	0.6	15.5	0.3	4.8	2.2	8.3	9.1	10.1	3.0	1.3
2014	7.6	1.8	8.2	5.2	3.6	0.8	3.1	1.9	8.9	1.1	1.6	2.2	0.7	15.9	0.2	5.0	2.4	7.8	8.8	9.1	2.9	1.2
2015	7.4	1.9	8.7	5.6	3.6	0.8	3.5	1.9	8.4	0.7	1.7	2.2	0.6	15.3	0.4	4.8	2.0	7.6	9.5	9.6	2.5	1.5
2016	7.2	2.0	8.8	5.5	3.5	1.0	3.4	2.0	8.8	0.7	1.7	2.3	0.7	15.8	0.3	4.7	2.1	7.2	9.3	9.2	2.6	1.2
2017	6.9	1.9	8.9	5.3	3.5	0.8	3.3	1.6	8.9	0.8	1.6	2.3	0.5	15.9	0.4	4.6	2.3	6.8	9.6	9.7	2.8	1.6
2018	7.2	1.7	8.7	5.5	3.7	0.9	3.5	2.1	9.3	0.8	1.8	2.2	0.6	15.2	0.4	4.9	2.2	6.8	9.5	9.3	2.5	1.3
2019	7.3	1.9	8.5	5.5	3.3	1.1	3.2	2.4	9.6	0.9	1.8	2.4	0.6	14.9	0.3	4.5	1.9	6.6	9.4	9.6	2.8	1.4
2020	7.3	1.9	9.0	5.6	3.6	0.8	3.5	2.6	9.9	1.1	1.5	2.3	0.8	14.3	0.4	4.8	1.8	6.3	9.6	8.8	2.8	1.5
2021	7.7	1.4	9.0	6.2	3.7	0.8	4.0	2.4	10.2	1.0	1.5	2.5	0.5	15.2	0.2	4.4	1.8	6.4	9.2	8.0	2.7	1.1
2022	7.5	1.6	9.6	5.6	3.5	0.8	3.2	2.5	10.9	0.8	1.6	2.5	0.7	14.1	0.3	4.2	2.0	6.0	9.2	8.7	3.2	1.3
2023	7.9	1.5	10.2	5.4	4.1	0.8	3.2	2.4	11.3	0.6	1.3	2.3	0.8	14.3	0.3	4.5	1.9	5.3	9.3	8.4	2.6	1.5

Supplementary table to Figure 2A, presenting the annual percentage distribution of each cancer type.

© 2026 Uozaki H et al. *JAMA Network Open*.

eTable 4. Age-Specific Cancer Type Composition

Age group	Other than the following	Multiple myeloma	Malignant lymphoma	Leukemia	Thyroid	Brain, nervous system	Kidney and other urinary organs	Bladder	Prostate	Ovary	Uterus	Breast	Skin	Lung, trachea	Larynx	Pancreas	Gallbladder and bile ducts	Liver	Colon/rectum	Stomach	Esophagus	Oral cavity and pharynx
Stillbirth	53.8	0.0	0.6	15.8	0.0	19.3	1.2	0.0	0.0	0.0	0.6	0.0	0.6	0.0	0.0	0.0	0.0	1.2	0.0	0.6	0.0	6.4
0–4yo	32.6	0.0	7.9	28.2	0.0	16.2	4.6	0.6	0.2	0.3	0.1	0.0	0.2	0.5	0.0	0.1	0.1	7.8	0.0	0.1	0.0	0.5
5–9yo	19.6	0.0	8.4	40.6	0.0	25.1	2.3	0.3	0.1	0.5	0.1	0.0	0.1	0.1	0.0	0.1	0.1	1.8	0.0	0.1	0.1	0.5
10–14yo	21.2	0.1	13.1	37.9	0.1	19.9	0.7	0.1	0.0	1.7	0.1	0.0	0.3	0.3	0.0	0.3	0.0	2.6	0.5	0.3	0.0	0.5
15–19yo	24.6	0.1	13.2	38.3	0.8	13.2	0.7	0.1	0.3	2.0	0.1	0.1	0.3	1.0	0.0	0.5	0.2	1.5	0.9	1.3	0.1	0.8
20–24yo	20.9	0.1	14.8	34.0	1.1	11.2	0.8	0.2	0.3	2.0	0.9	0.2	0.9	1.5	0.0	0.7	0.3	2.1	1.9	4.2	0.1	1.6
25–29yo	17.7	0.3	11.7	27.9	1.4	9.0	0.9	0.2	0.2	2.6	2.4	1.4	1.0	3.1	0.0	1.2	0.7	3.3	3.6	9.7	0.1	1.5
30–34yo	12.8	0.4	10.0	23.5	1.9	7.3	1.0	0.4	0.2	2.3	3.0	3.6	1.0	5.1	0.1	1.9	0.9	4.7	4.7	13.5	0.3	1.5
35–39yo	9.4	0.6	8.5	17.5	2.3	6.0	1.3	0.4	0.2	2.6	3.6	5.0	0.9	7.9	0.1	2.6	1.5	6.5	5.6	15.1	0.7	1.6
40–44yo	7.3	1.0	7.8	13.4	2.5	4.1	1.8	0.6	0.3	3.1	3.6	5.1	0.7	9.7	0.1	3.7	1.7	9.6	6.0	14.6	1.4	1.8
45–49yo	6.0	1.4	6.8	9.8	2.8	3.2	2.0	0.7	0.5	3.1	3.8	5.1	0.5	10.8	0.2	4.4	2.2	13.2	6.0	12.9	2.6	2.0
50–54yo	5.2	1.6	6.0	7.4	2.8	2.1	2.4	0.9	0.6	2.8	3.4	4.2	0.5	11.8	0.3	4.9	2.4	16.6	6.3	12.0	3.7	2.1
55–59yo	4.9	1.8	5.8	5.9	2.6	1.6	2.6	1.1	1.3	1.9	2.8	3.1	0.4	13.4	0.4	5.4	2.8	17.9	6.4	11.9	4.2	2.0
60–64yo	4.6	1.8	5.6	5.0	2.6	1.2	2.7	1.3	2.1	1.5	2.3	2.2	0.4	15.6	0.4	5.5	3.2	16.5	6.8	12.2	4.4	1.9
65–69yo	4.3	1.9	5.5	4.3	2.6	1.0	2.7	1.7	3.4	1.0	1.9	1.7	0.4	17.7	0.5	5.5	3.6	14.2	7.3	13.0	4.0	1.8
70–74yo	4.2	1.9	5.6	3.8	2.5	0.8	2.8	2.1	5.0	0.9	1.7	1.4	0.4	18.8	0.5	5.7	4.1	11.5	7.7	13.4	3.6	1.5
75–79yo	4.3	1.7	5.9	3.4	2.6	0.5	2.8	2.4	6.8	0.8	1.6	1.3	0.5	18.4	0.5	5.4	4.6	9.3	8.6	14.1	3.0	1.4
80–84yo	4.0	1.5	6.0	2.9	2.8	0.4	2.9	2.9	8.9	0.6	1.5	1.4	0.7	16.5	0.5	5.5	5.1	7.6	9.9	14.5	2.6	1.2
85–89yo	4.2	1.3	6.2	2.4	3.1	0.4	3.2	3.2	10.8	0.6	1.4	1.6	0.9	14.5	0.4	5.2	5.0	6.0	11.6	15.2	1.9	1.2
90–94yo	4.0	0.9	5.3	1.7	3.3	0.4	3.2	3.1	11.9	0.6	1.8	2.0	1.0	12.9	0.4	4.9	5.4	5.0	14.2	15.5	1.5	1.0
95–99yo	3.7	1.0	4.6	1.8	4.1	0.6	3.9	3.1	11.5	0.5	1.9	3.5	1.5	12.0	0.2	4.4	5.0	4.3	15.9	14.2	1.4	0.8
100yo–	4.3	0.6	4.8	0.6	4.8	0.0	3.1	2.8	9.1	0.9	2.0	6.3	2.6	9.4	0.3	4.0	4.0	2.3	22.2	14.2	0.9	1.1

Supplementary table to Figure 2B, presenting the percentage distribution of each cancer type across age groups.

© 2026 Uozaki H et al. *JAMA Network Open*.

eTable 5. Cancer Types by Sex

	Male	Female
Other than the following	5.1	5.3
Multiple myeloma	1.4	2.0
Malignant lymphoma	5.9	6.5
Leukemia	5.6	6.5
Thyroid	1.8	4.2
Brain, nervous system	1.5	2.0
Kidney and other urinary organs	2.9	2.1
Bladder	2.0	1.2
Prostate	6.4	0.0
Ovary	0.0	4.0
Uterus	0.0	6.4
Breast	0.0	6.5
Skin	0.5	0.6
Lung, trachea	17.9	10.4
Larynx	0.6	0.1
Pancreas	4.8	5.8
Gallbladder and bile ducts	2.6	5.6
Liver	13.7	8.6
Colon/rectum	7.4	8.2
Stomach	13.7	11.7
Esophagus	4.2	1.3
Oral cavity and pharynx	1.9	1.1

Supplementary table for Figure 2C. The proportions of each cancer type in each sex are shown as percentages.

© 2026 Uozaki H et al. *JAMA Network Open*.

eTable 6. Cancer Types in Single vs Multiple Primary Cancers

	Single cancer	Double cancer	Triple or more cancer
Other than the following	5.6	4.1	3.8
Multiple myeloma	1.8	1.1	0.8
Malignant lymphoma	6.7	4.5	3.6
Leukemia	6.8	3.2	2.1
Thyroid	1.5	6.2	5.2
Brain, nervous system	2.0	0.5	0.3
Kidney and other urinary organs	2.2	3.8	4.7
Bladder	1.5	2.5	3.5
Prostate	2.7	9.1	9.9
Ovary	1.5	0.8	0.4
Uterus	2.1	2.2	1.6
Breast	2.1	2.5	2.3
Skin	0.5	0.6	0.9
Lung, trachea	15.9	13.8	13.9
Larynx	0.3	0.7	1.1
Pancreas	5.7	3.6	2.8
Gallbladder and bile ducts	4.0	2.5	1.9
Liver	13.4	8.3	5.8
Colon/rectum	6.4	10.5	14.7
Stomach	12.7	14.3	13.9
Esophagus	3.1	3.4	4.0
Oral cavity and pharynx	1.5	1.9	2.6

Supplementary table for Figure 2D, showing the percentage distribution of each cancer type in single and multiple cancers.

© 2026 Uozaki H et al. *JAMA Network Open*.

eTable 7. Changes in ERA Rankings for Cancers by Decade

	1960s	1970s	1980s	1990s	2000s	2010s	2020s[a]
1	BrainNS 34.2[b]	Thyroid 17.7	Thyroid 13.2	Thyroid 15.1	Thyroid 15.6	Thyroid 18.4	Thyroid 21.2
2	Myeloma 10.9	BrainNS 11.6	BrainNS 10.4	BrainNS 8.2	BrainNS 6.2	Prostate 6.9	Prostate 8.5
3	Lymphoma 8.2	Lymphoma 10.1	Lymphoma 5.8	Lymphoma 5.5	Lymphoma 5.7	Lymphoma 6.4	Lymphoma 7.0
4	Thyroid 8.2	Leukemia 7.2	Leukemia 5.6	Leukemia 5.4	Leukemia 5.3	Leukemia 5.8	Leukemia 6.3
5	Leukemia 7.8	KidneyU 7.0	KidneyU 5.3	Skin 4.8	Prostate 5.3	BrainNS 4.7	Skin 4.4
6	KidneyU 7.2	Prostate 6.2	Prostate 4.8	Prostate 4.7	Skin 4.4	Skin 4.6	Larynx 4.2
7	OralCP 6.5	Myeloma 5.1	Myeloma 4.5	KidneyU 4.1	Myeloma 3.7	Myeloma 4.5	Myeloma 4.1
8	GBBD 5.9	Skin 4.4	Skin 4.3	Myeloma 3.7	KidneyU 3.5	KidneyU 3.3	BrainNS 3.7
9	LungT 4.9	OralCP 4.1	OralCP 4.1	OralCP 3.0	Larynx 2.8	Larynx 3.3	KidneyU 3.3
10	Ovary 4.8	Liver 4.0	Liver 3.2	Liver 2.9	Liver 2.4	Liver 2.5	Bladder 2.8
11	Prostate 4.1	Bladder 3.6	Bladder 3.1	Larynx 2.8	Bladder 2.4	Uterus 2.4	Esophagus 2.7
12	Pancreas 3.9	Ovary 3.4	Ovary 2.7	Bladder 2.8	Esophagus 2.1	Bladder 2.3	Liver 2.7
13	Breast 3.5	LungT 3.4	LungT 2.6	Uterus 2.4	Uterus 2.1	Esophagus 2.2	Uterus 2.2
14	Bladder 3.5	Esophagus 3.1	Esophagus 2.6	Esophagus 2.3	OralCP 1.9	LungT 2.0	Stomach 2.2
15	Larynx 2.7	Pancreas 3.0	Pancreas 2.5	LungT 2.1	LungT 1.9	Stomach 1.9	Ovary 2.1
16	Esophagus 2.5	Larynx 2.8	Larynx 2.3	Ovary 2.0	Ovary 1.8	OralCP 1.9	LungT 1.8
17	Uterus 2.1	Uterus 2.5	Uterus 2.3	Pancreas 2.0	Pancreas 1.7	Ovary 1.8	OralCP 1.8
18	Skin 1.8	GBBD 2.4	GBBD 2.3	Breast 1.8	Stomach 1.6	ColonR 1.7	ColonR 1.8
19	Liver 1.8	Breast 2.0	Breast 2.2	ColonR 1.5	ColonR 1.5	Breast 1.5	Breast 1.6
20	ColonR 1.8	ColonR 1.8	ColonR 1.7	GBBD 1.5	Breast 1.3	Pancreas 1.4	Pancreas 1.2
21	Stomach 1.4	Stomach 1.4	Stomach 1.4	Stomach 1.5	GBBD 1.3	GBBD 1.1	GBBD 1.1

a: 2020–2023, b: ERA.

Abbreviations: LungT: Lung, trachea, ColonR: Colon/rectum, BrainNS: Brain, nervous system, KidneyU: Kidney and other urinary organs, GBBD: Gallbladder and bile ducts, OralCP: Oral cavity and pharynx.

© 2026 Uozaki H et al. *JAMA Network Open*.

eTable 8. Latent Cancer and Metastasis in Autopsies (1986–2023) (*N*=811,159)

	Latent cancer	Metastasis (LN, organ)	(rate)
Prostate	14,777	420 (120, 378)	(2.8%)
Thyroid	8,197	451 (277, 222)	(5.5%)
Lung, trachea	2,861	478 (335, 302)	(16.7%)
Kidney and other urinary organs	2,212	65 (15, 60)	(2.9%)
Colon/rectum	2,094	160 (78, 122)	(7.6%)
Liver	1,548	125 (37, 112)	(8.1%)
Stomach	1,275	159 (123, 108)	(12.5%)
Pancreas	617	169 (109, 140)	(27.4%)
Gallbladder and bile ducts	430	93 (57, 71)	(21.6%)
Bladder	296	24 (8, 24)	(8.1%)
Malignant lymphoma	250	135 (87, 120)	(54.0%)
Esophagus	244	38 (30, 23)	(15.6%)
Uterus	196	20 (7, 18)	(10.2%)
Multiple myeloma	183	58 (23, 54)	(31.7%)
Breast	94	15 (11, 10)	(16.0%)
Ovary	87	17 (8, 15)	(19.5%)
Leukemia	72	35 (14, 35)	(48.6%)
Oral cavity and pharynx	59	12 (9, 8)	(20.3%)
Larynx	30	1 (0, 1)	(3.3%)
Brain, nervous system	24	6 (0, 6)	(25.0%)
Skin	12	1 (1, 1)	(8.3%)
Others	575	167 (95, 110)	(29.0%)
Total	36,133	2,649 (1,444, 1,940)	(7.3%)

From 1986 to 2023, 811,159 individuals were registered, including 519,026 men, 290,677 women, and 1,456 of unspecified sex.

© 2026 Uozaki H et al. *JAMA Network Open*.

eTable 9. Composition of Latent Cancer by Sex

	Male	Female
Other than the following	1.4	2.3
Multiple myeloma	0.2	0.3
Malignant lymphoma	0.4	0.7
Leukemia	0.6	1.0
Thyroid	14.8	50.2
Brain, nervous system	0.0	0.1
Kidney and other urinary organs	6.1	6.2
Bladder	0.8	0.9
Prostate	52.6	0.0
Ovary	0.0	1.1
Uterus	0.0	2.4
Breast	0.0	1.1
Skin	0.0	0.0
Lung, trachea	7.1	10.7
Larynx	0.1	0.0
Pancreas	1.5	2.5
Gallbladder and bile ducts	0.9	2.2
Liver	4.2	4.6
Colon/rectum	5.0	8.6
Stomach	3.3	4.4
Esophagus	0.8	0.4
Oral cavity and pharynx	0.2	0.1

Supplementary table for Figure 4C. The table shows, for each sex, the percentage distribution of each cancer type among latent cancers.

© 2026 Uozaki H et al. *JAMA Network Open*.
