# Supplementary material for: Trends in the Hidden Burden of Cancer in an Autopsy-Based Study Over 66 Years in Japan
Source: JAMA Netw Open. 2026 Feb 5;9(2):e2557812. doi: 10.1001/jamanetworkopen.2025.57812 (PMC12878435; doi:10.1001/jamanetworkopen.2025.57812)
Supplement: Supplement 2. — Data Sharing Statement [file jamanetwopen-e2557812-s002.pdf]

Data Sharing Statement

Data
Data available: Yes
Data types: Deidentified participant data
How to access data: All data used in this study are publicly available as books, APAC-J by JSP. Some of the data is publicly accessible at the website of JSP in Japanese (https://pathology.or.jp/kankoubutu/autopsy-index.html) (accessed on August 4, 2025).
When available: With publication

Supporting Documents
Document types: None

Additional Information
Who can access the data: Researchers whose proposed use of the data has been approved.
Types of analyses: for any purpose
Mechanisms of data availability: after approval of a proposal
